# Diversity of late Neogene Monachinae (Carnivora, Phocidae) from the North Atlantic, with the description of two new species

**DOI:** 10.1098/rsos.172437

**Published:** 2018-03-07

**Authors:** Leonard Dewaele, Carlos Mauricio Peredo, Pjotr Meyvisch, Stephen Louwye

**Affiliations:** 1 Department of Geology, Ghent University, Ghent, Belgium; 2 Directorate ‘Earth and History of Life’, Royal Belgian Institute of Natural Sciences, Brussels, Belgium; 3 Department of Environmental Science and Policy, George Mason University, Fairfax, VA, USA; 4 Department of Paleobiology, Smithsonian National Museum of Natural History, Washington, DC, USA

**Keywords:** Neogene, biodiversity, North Atlantic, Phocidae, Monachinae

## Abstract

While the diversity of ‘southern seals’, or Monachinae, in the North Atlantic realm is currently limited to the Mediterranean monk seal, *Monachus monachus*, their diversity was much higher during the late Miocene and Pliocene. Although the fossil record of Monachinae from the North Atlantic is mainly composed of isolated specimens, many taxa have been erected on the basis of fragmentary and incomparable specimens. The humerus is commonly considered the most diagnostic postcranial bone. The research presented in this study limits the selection of type specimens for different fossil Monachinae to humeri and questions fossil taxa that have other types of bones as type specimens, such as for *Terranectes parvus*. In addition, it is essential that the humeri selected as type specimens are (almost) complete. This questions the validity of partial humeri selected as type specimens, such as for *Terranectes magnus*. This study revises *Callophoca obscura*, *Homiphoca capensis* and *Pliophoca etrusca*, all purportedly known from the Lee Creek Mine, Aurora, North Carolina, in addition to their respective type localities in Belgium, South Africa and Italy, respectively. *C. obscura* is retained as a monachine seal taxon that lived both on the east coast of North America and in the North Sea Basin. However, *H. capensis* from North America cannot be identified beyond the genus level, and specimens previously assigned to *Pl. etrusca* from North America clearly belong to different taxa. Indeed, we also present new material and describe two new genera of late Miocene and Pliocene Monachinae from the east coast of North America: *Auroraphoca atlantica* nov. gen. et nov. sp., and *Virginiaphoca magurai* nov. gen. et nov. sp. This suggests less faunal interchange of late Neogene Monachinae between the east and west coasts of the North Atlantic than previously expected.

## 1. Introduction

Among the semi-aquatic carnivoran clade of Pinnipedia, the family of true seals, or Phocidae, are remarkable for their biogeography. Phocidae are usually subdivided into two subfamilies, the ‘southern seals’, or Monachinae; and the ‘northern seals’, or Phocinae, see [1]. This reflects the current biogeography of both subfamilies, in which extant Monachinae include the Caribbean, Hawaiian and Mediterranean monk seals, the elephant seals along the eastern shores of the Pacific Ocean, and the elephant seals, leopard seal (*Hydrurga leptonyx* de Blainville, 1820), Weddell seal (*Leptonychotes weddellii* Lesson, 1826), crabeater seal (*Lobodon carcinophaga* Hombron and Jacquinot, 1842) and Ross seal (*Ommatophoca rossii* Gray, 1844) in the Antarctic and sub-Antarctic waters, which all live southerly of the ‘northern’ Phocinae. Phocinae, on the other hand, are largely restricted to the North Atlantic and North Pacific oceans and the Arctic Ocean. However, the fossil record from the late Neogene shows that ‘southern seals’ were much more common and diverse in the North Atlantic realm than they are today [2–4].

Molecular evidence indicates that stem phocids diverged from other pinnipeds around 23 Ma, at the Oligocene–Miocene boundary, but crown phocids, comprising the two subfamilies Monachinae and Phocinae, diverge in the middle Miocene around 16 Ma [5]. Notwithstanding the molecular inferences, two Monachinae, *Afrophoca libyca* Koretsky and Domning, 2014 and *Monotherium gaudini* (Guiscardi, 1870)*,* known only from cranial elements ([6,7]; L. Dewaele 2017, personal observation), are older than 16 Ma: *Af. libyca* from Libya is Burdigalian in age (early Miocene, *ca* 19 Ma) [6] and *M. gaudini* from Italy may range from the Chattian (late Oligocene) to the Aquitanian (early Miocene) ([8,9]; L. Dewaele 2017, personal observation); therefore, neither is within the scope of this study and they will not be discussed in this study. Throughout the middle and late Miocene and early Pliocene, phocid taxa underwent (at least) one dispersal event across the North Atlantic Ocean: *Leptophoca proxima* (Van Beneden, 1876) and *Monotherium aberratum* Van Beneden, 1876 from the early-to-middle Miocene and late Miocene, respectively, are known from both sides of the North Atlantic [10,11], and many of the extinct phocid taxa described from the late Miocene and early Pliocene of the southern North Sea Basin by Van Beneden [2,3] have also been identified in the early Pliocene of North Carolina, USA [4]. However, subtle differences between specimens identified as the same taxon from across the Atlantic have led authors to code them as separate operational taxonic units (OTUs) in phylogenetic analyses [12]. Moreover, not all extinct phocid species have been found on both sides of the Atlantic, notably the enigmatic *Nanophoca vitulinoides* (Van Beneden, 1871) from the southern North Sea Basin [13].

Therefore, a revision of the different phocid OTUs from the North Atlantic realm is required in order to obtain a more conclusive view on their diversity and palaeobiogeography. The abundance of monachines in the North Atlantic temperate latitudes during the late Miocene–early Pliocene is particularly interesting given that they are conspicuously absent from this region today; modern monachines are restricted to the Mediterranean and Caribbean seas, Hawaii and the eastern Pacific and Antarctic and sub-Antarctic waters (e.g. [1,14]).

In this study, we reassess the humeri of the three taxa of monachine seals from the Neogene of the North Atlantic realm: *Callophoca obscura* Van Beneden, 1876, *Homiphoca capensis* (Hendey and Repenning, 1972) and *Pliophoca etrusca* Tavani, 1941 [2–4,12,15]*.* Humeri are selected and considered for two reasons. First, humeri are considered the most diagnostic postcranial bones in Phocidae[16–18]. Given the overall scarcity of more diagnostic cranial specimens, a taxonomy based on the most complete humeri possible is required to properly identify and compare phocid taxa. Second, due to their robust nature, humeri are among the most commonly preserved fossil phocid bones [4]. This is clearly advantageous over using less diagnostic types of bone that are more rarely preserved in the fossil record.

*Messiphoca mauretanica* Muizon, 1981 from the Messinian (upper Miocene) of Algeria [19] and *Terranectes* spp. from the upper Miocene of Maryland, USA [20] have formerly been identified as Monachinae from the North Atlantic realm, with a known humerus, but they are problematic for various reasons. *M. mauretanica* is represented by an associated left humerus, left ulna, left radius and six dorsal vertebrae (MNHN.F.ORN1, holotype) [19]. An isolated partial cranium (MNHN.F.ORN2, paratype) has been described as well. To date, no other specimens of *M. mauretanica* are known. Given the overall relatively poor quality of the small fossil record of *M. mauretanica*, this taxon needs a formal revision, pending the discovery of more complete specimens, i.e. more complete humeri, prior to further research involving this taxon. Nevertheless, in this study, *M. mauretanica* will be considered for comparison purposes because of the association of multiple bones in the holotype (MNHN.F.ORN1).

The genus *Terranectes* Rahmat, Koretsky, Osborne, Alford, 2017 was recently described, based on isolated material from the Chesapeake Bay Area, Maryland, USA [20]. Issues regarding the incomparability of both species to each other and to other known taxa make comparison impossible. Rahmat *et al*. [20] invoke Koretsky's [16] ecomorphotype hypothesis to support the designation of isolated specimens of different types of bone to different taxa. Koretsky's [16] ecomorphotype hypothesis is constructed for mandibles, humeri and femora. Currently, this hypothesis has been inadequately tested to be considered as a diagnostic tool, especially with regard to postcranial bones other than humeri and femora, which have not been considered in the original study by Koretsky [16]. For *Terranectes magnus* Rahmat, Koretsky, Osborne, Alford, 2017, the holotype specimen CMM-V-4710, is a partial humerus. However, although humeri are usually considered the most diagnostic postcranial bones in Phocidae, the holotype specimen of *T. magnus* is very incomplete, inhibiting proper comparison with other taxa (see below). Therefore, we judge that there is no scientific basis to support the assumption that the isolated specimens of both *T. magnus* and *Terranectes parvus* Rahmat, Koretsky, Osborne, Alford, 2017 indeed belong to the same genus. Pending future discoveries, the genus *Terranectes* should be considered a nomen dubium.

We also present new fossil phocid material from the east coast of North America, dredged off the Nottoway River bed west of Franklin, Virginia, USA. Previous research showed that the Chowan River tributaries, including the Nottoway and Meherrin rivers yield a large number of vertebrate fossils, including the type and only known specimen from the inoid odontocete *Meherrinia isoni* Geisler, Godfrey, Lambert, 2012.

## 2. Method and materials

### 2.1. Nomenclature

To maintain consistency with recent publications on extinct Phocidae, we follow the nomenclature and terminology used by Dewaele *et al*. [11,13] for phocid humeral anatomy where possible, and otherwise follow that of Evans & Lahunta [21] for the domestic dog.

### 2.2. Institutional abbreviations

CMM, Calvert Marine Museum, Solomons, Maryland, USA; IRSNB, Institut Royal des Sciences Naturelles, Brussels, Belgium; MNHN, Muséum National d'Histoire Naturelle, Paris, France; MSNUP, Museo di Storia Naturale, Università di Pisa, Pisa, Italy; SAM, South African Museum, Iziko Museums of South Africa, Cape Town, South Africa; USNM, Department of Paleobiology, National Museum of Natural History, Washington, DC, USA.

### 2.3. Fossil specimen sample

This study includes all late Miocene–early Pliocene Monachinae from the North Atlantic realm that are known from substantial material in the fossil record: *C. obscura*, *H. capensis* and *Pliophoca etrusca*. Comparison material also includes other Neogene Monachinae *Acrophoca longirostris* Muizon, 1981, *Australophoca changorum* Valenzuela-Toro, Pyenson, Gutstein, Suárez, 2016, *Piscophoca pacifica* Muizon 1981 and *Properitpychus argentinus* (Ameghino, 1893), all from the Southern Hemisphere. These comparisons are based on personal observations (*Ac. longirostris* and *Pi. pacifica*) and bibliographic data (*Ac. longirostris*, *Au. changorum*, *Pi. pacifica* and *Pr. argentinus*) [22–24].

### 2.4. Intra- and interspecific long bone variation

**Figure 1.**
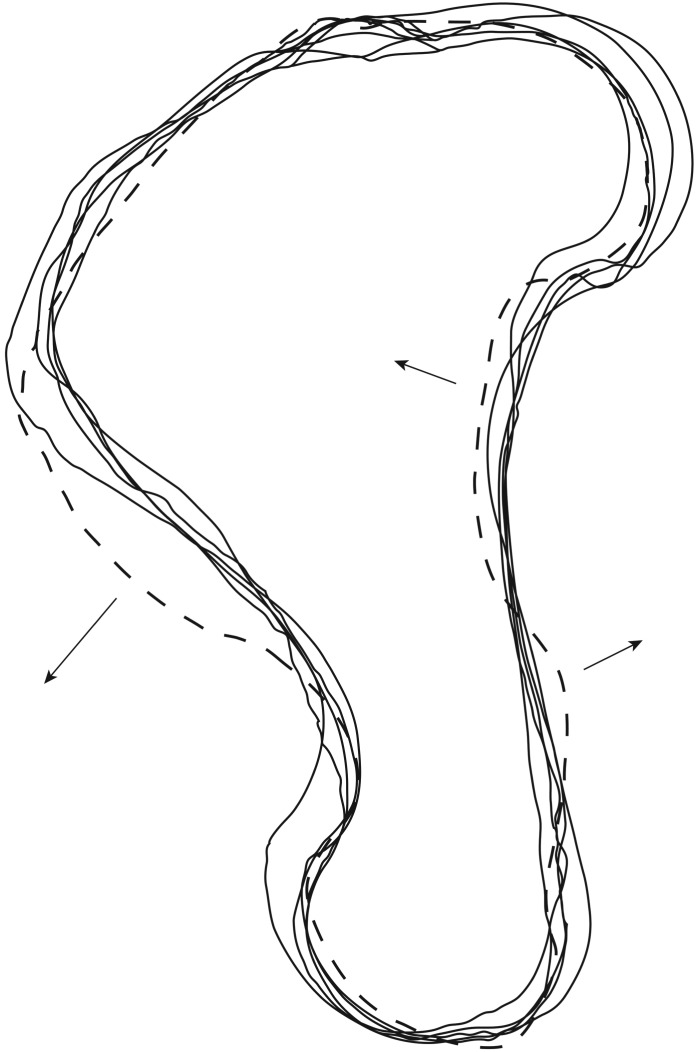
Line drawings showing the basic morphological differences in the humeri of two different genera of extant Monachine. Five specimens of the leopard seal (*Hydrurga leptonyx*) indicated by full lines and one specimen of the Weddell seal (*Leptonychotes weddellii*) indicated by a dashed line. All specimens rescaled to the same proximodistal size. Arrows indicate the most important differences in the humerus between both genera.

Understanding intra- and interspecific variation in long bone shape among Monachinae is crucial to assess the degree of completeness required for an isolated fossil humerus to be considered diagnostic. A detailed study on intra- and interspecific monachine humerus shape is beyond the scope of this study. However, preliminary qualitative observations suggest that complete or nearly complete humeri can be treated as diagnostic bones to differentiate between different species of Monachinae. A very basic qualitative comparison of four humeri of (both sexes of) *Hydrurga leptonyx* and one humerus of *Leptonychotes weddellii* suggests that humeri are sufficiently diagnostic to distinguish taxa when complete (figure 1). This figure shows that the four complete humeri of *H. leptonyx* differ relatively little from one another, while the humerus of *L. weddellii* differs from the humeri of *H. leptonyx* on key morphological features, such as the curvature of the diaphysis and the morphology of the distal portion of the deltopectoral crest. However, the number of morphologically varying characters is limited, suggesting that complete or nearly complete humeri are required to differentiate between closely related species. All characters listed in the diagnoses of taxa below are considered to represent interspecific variation, intraspecific variation being discarded based on personal quantitative observations of extant Monachinae.

## 3. Geological background and dinoflagellate cyst biostratigraphy

### 3.1. Callophoca obscura

*Callophoca obscura* is known from the ‘Scaldisian’ of the southern margin of the North Sea Basin, in Belgium, and of the Yorktown Formation in the Lee Creek Mine, Aurora, North Carolina, USA [2–4]. However, as mentioned above, the Scaldisian is currently considered an obsolete term and its use has been discontinued [25]. Different authors assign different ages and stratigraphic intervals to the Scaldisian ([25]: table 1), but in general, it is considered that the Scaldisian is roughly equivalent to the early Pliocene Kattendijk Formation [26–28]. However, many phocid fossils from the ‘Scaldisian’ show signs of reworking [13]. Most of the vertebrate remains recovered from the Kattendijk Formation come from the basal gravel [29], suggesting reworking of many of the specimens from the underlying Deurne Sands Member of the Diest Formation (late Miocene) or from the latest Miocene–earliest Pliocene depositional hiatus between the Diest and Kattendijk formations. The recent redescription of *Nanophoca vitulinoides* showed that this taxon actually lived during the Miocene and that most specimens of *N. vitulinoides* are Miocene in age but reworked into the early Pliocene basal gravel of the Kattendijk Formation [13]. Hence, the historical age determinations should be considered with care. For the Yorktown Formation, Ward & Blackwelder [30] state a Zanclean (early Pliocene) age for the formation in the Chesapeake Bay. Radiometric dating of the Rushmere Member, the second oldest member of the Yorktown Formation, returned an age of 4.4 ± 0.2 Ma [31]. They also considered the basal strata of the Yorktown Formation, the Sunken Meadow Member, equivalent to foraminiferal zone D18, with an onset at 5.0 Ma, unconformably overlying the 6.46 Ma upper strata of the Eastover Formation.

**Table 1.** Measurements (in mm) of the holotype humeri of *Auroraphoca atlantica* and *Virginiaphoca magurai*. Measurements were taken to the nearest 0.1 mm using an analogous caliper. Measurement approach follows Koretsky [1].

	*Auroraphoca atlantica*	*Virginiaphoca magurai*
	USNM 181419	USNM 639750
total length	159.3	134.0
length deltopectoral crest	108.2	86.4
height head	38.9	28.9
height trochlea	22.1	18.9
width head	44.3	40.1
width deltopectoral crest	29.6	25.2
width proximal epiphysis	62.5	56.5
width distal epiphysis	66.6	54.8
distal width trochlea	39.6	34.2
anterior width trochlea	26.2	24.2
transverse width mid-diaphysis	28.5	24.6
anteroposterior thickness proximal epiphysis	74.0	61.4
anteroposterior thickness medial condyle	32.0	25.9
anteroposterior thickness lateral condyle	34.2	26.9
diameter mid-diaphysis and deltopectoral crest in lateral view	58.0	49.3

Two sediment samples (sample L15–1105 from the large (possible male) humerus of *C. obscura*, IRSNB M1156, originally described as *Mesotaria ambigua* Van Beneden, 1876 and sample L15–1108 from a small (possible female) humerus of *C. obscura*, IRSNB 1214) recovered from bone cavities were palynologically analysed for organic-walled dinoflagellate cysts (dinocysts) and acritarchs (electronic supplementary material). Unfortunately, the revision of *C. obscura* humeri proved that the latter specimen cannot be assigned to the species unambiguously. Both sediment samples return a Messinian age (latest Miocene), which can be regarded as a minimum age of the *C. obscura* specimens. However, because reworking of dinoflagellate cysts has been detected, it is impossible to elucidate an absolute age interval.

### 3.2. Homiphoca capensis

An age of 5.15 ± 0.1 Ma (earliest Zanclean, earliest Pliocene) had been proposed for the Langeberg quartzose sand and Muishond Fontein peletal phosphorite members of the South African Varswater Formation, containing virtually all South African *Homiphoca capensis* specimens ([32–34], and references therein). A number of phocid specimens from the Yorktown Formation at Lee Creek Mine, Aurora, North Carolina have been assigned to *H. capensis* [4], despite the strong geographical distance between the east coast of North America and the only other known (type) locality of *H. capensis*, the ‘E’ Quarry at Langebaanweg, northwest of Cape Town, South Africa. Others (C. de Muizon 2017, personal communication) disagree with the identification of *H. capensis* from North America. Nevertheless, the age of the Yorktown Formation roughly corresponds with the Langeberg Quartzose Sand Member (LQSM) and the Muishond Fontein Peletal Phosphorite Member (MPPM) from South Africa. The latter centre around 5.15 Ma [34], while Ward & Blackwelder [30] show that the basal Sunken Meadow Member of the Yorktown Formation has been dated to 5.0 Ma using foraminifera biostratigraphy, and Akers [31] presented a radiometrically measured age of 4.4 ± 0.2 Ma for the overlying Rushmere Member of the Yorktown Formation. Despite the difference, LQSM, MPPM and the Yorktown Formation are entirely Zanclean in age.

### 3.3. Pliophoca etrusca

From Italy, only the type specimen of *Pliophoca etrusca* is known. Tavani [35] assigned multiple other isolated and associated bones to *Pl. etrusca*, but Berta *et al*. [12] attributed these to *Pliophoca* cf. *Pl. etrusca*. A Piacenzian age (late Pliocene) has been assigned to the holotype skeleton: the layer of the Pliocene Argille Azzurre Formation it came from has been dated to 3.19–2.82 Ma [12,36]. The geological and historical background of the North American specimens of ‘*Pliophoca etrusca*’ is identical to the background of the North American specimens of *H. capensis*: *Pl. etrusca* from North America is only known from the Yorktown Formation at the Lee Creek Mine, which had been dated to the Zanclean (early Pliocene) (see above).

### 3.4. *Auroraphoca atlantica* nov. gen. et nov. sp.

Two humeri that were previously assigned to *Pliophoca etrusca* from North America (USNM 181419 and USNM 250290) [4] have both been recovered from the Lee Creek Mine in Aurora, Beaufort County, North Carolina. As has been noted above, the fossiliferous stratum at the Lee Creek Mine is the early Pliocene Yorktown Formation, with the basal Sunken Meadow Member corresponding with the 5.0 Ma foraminiferal zone D18 and the overlying Rushmere Member radiometrically dated to 4.4 ± 0.2 Ma [30,31] (see above).

### 3.5. *Virginiaphoca magurai* nov. gen. et nov. sp.

**Figure 2.**
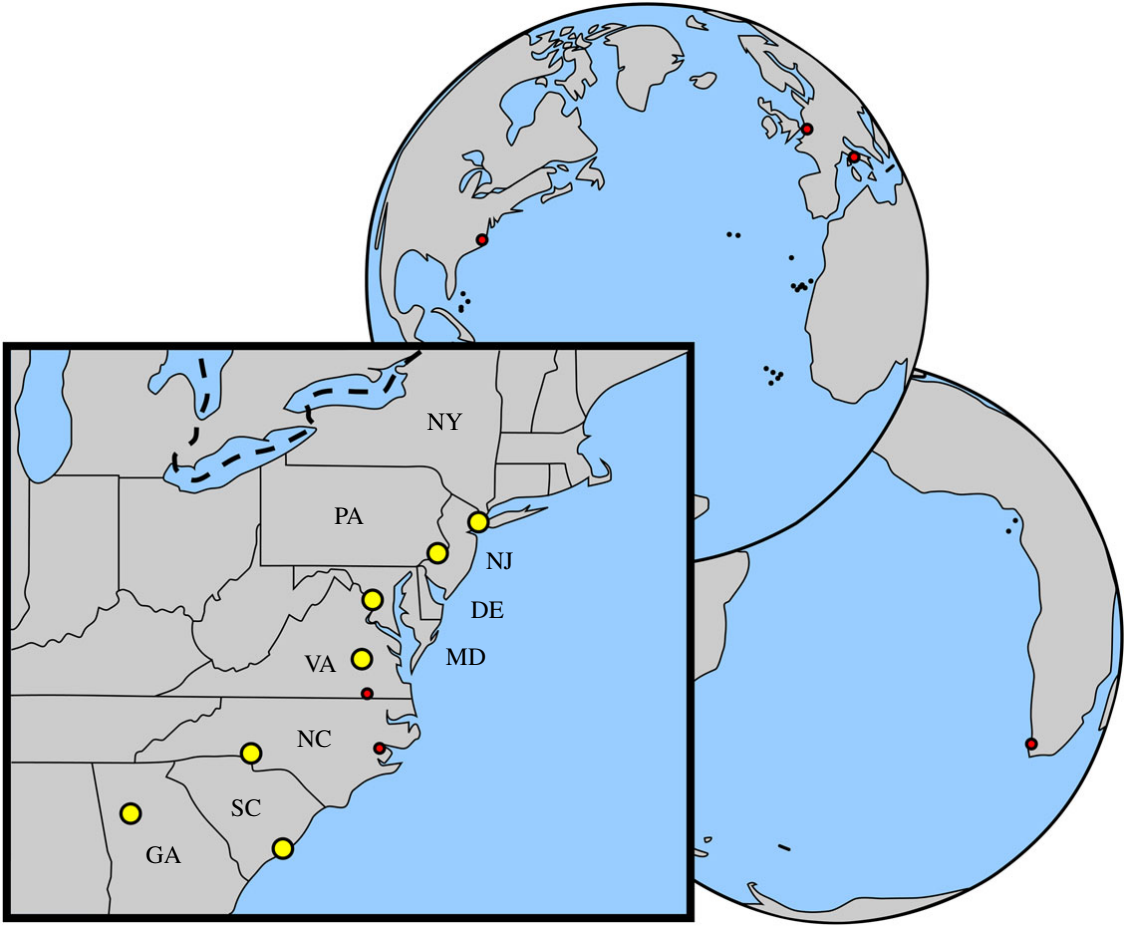
Map showing the geographical distribution of all Monachinae covered in this study. Localities are indicated by red dots: *Callophoca obscura* from Belgium, *Pliophoca etrusca* from Italy, *Homiphoca* sp. in South Africa; *Auroraphoca atlantica* nov. gen. et nov. sp., *C. obscura* and *Homiphoca* sp. in Aurora, North Carolina and *V. magurai* nov. gen. et nov. sp. near Franklin, Virginia. DE, Delaware; GA, Georgia; MD, Maryland; NC, North Carolina; NJ, New Jersey; NY, New York; PA, Pennsylvania; SC, South Carolina; VA, Virginia. Dashed line indicates border between Canada and the United States of America. Yellow dots indicate major cities (from east to west: Atlanta, Georgia; Raleigh, North Carolina; Charleston, South Carolina; Charlotte, Virginia; Washington, DC; Philadelphia, Pennsylvania; New York, New York).

One newly described humerus (USNM 639750) that has been dredged off the Nottoway River bed at Franklin, Virginia, is assigned to the new taxon *Virginiaphoca magurai*. The holotype and only known specimen, humerus USNM 639750, was found *ex situ* by Joseph Magura and was ‘clean’, i.e. no original sediment had been attached to the specimens that could be used in elucidating its lithostratigraphic or biostratigraphic origin. However, the specimen shows only few signs of rolling, and hence, fluviatile transport of the specimen must have been limited. The possible stratigraphic provenance covers the upper Miocene Cobham Bay Member (CBM) of the Eastover Formation and the lower Pliocene Yorktown Formation (figure 2), because both outcrop in the river banks and riverbed of the Nottoway River in the Franklin area [30]. A similar strategy had been employed to ‘date’ the fossils of the river dolphin *Meherrinia isoni* from the nearby Meherrin River in North Carolina [37]. The Yorktown Formation is dated to the Zanclean [30]. Ward & Blackwelder [30] also presented an age of 8.7 ± 0.4 Ma for the lower levels of the CBM, and 6.46 ± 0.15 Ma for subsurface sampling of this member, based on radiometric dating of glauconite. Hence, the CBM is of late Tortonian to Messinian age (late Miocene). Although colour of a fossil bone is only a weak basis to support correlation with a given stratum, specimen USNM 639750 is dark grey with orange stains. And while the majority of the bones from the Yorktown Formation in the Lee Creek Mine are more yellow to beige in colour, Ward & Blackwelder [30, p. 20] argue that the CBM is ‘Grayish blue (5 PB 5/2) where fresh, the Cobham Bay sediments weather to a yellowish orange (10 YR 8/6).’ This description of the colour of the CBM corresponds with the observed colour pattern of specimen USNM 639750. Currently, no specimens that have been recovered from the Yorktown Formation at the Lee Creek Mine can be attributed to *V. magurai*. Although the latter does not prove the absence of the species throughout the entire Yorktown Formation elsewhere, it supports the claim that the CBM is the more likely origin of the type specimen.

**Systematic palaeontology**

**Phocidae** Gray, 1821

**Monachinae** Gray, 1869

Genus ***Callophoca*** Van Beneden, 1876

**Type and only included species.** *Callophoca obscura* Van Beneden, 1876

**Diagnosis.** As for the species.

**Figure 3.**
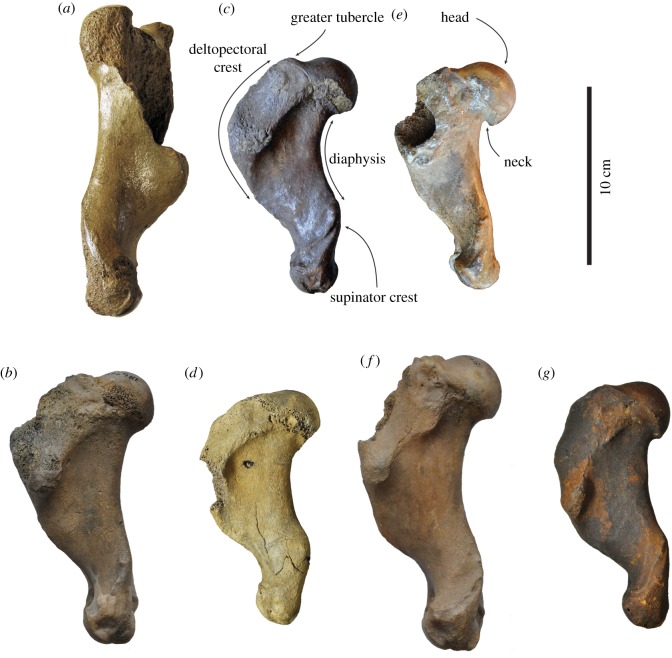
Comparison of humeri in lateral view. Right lectotype humerus IRSNB 1156-M177 (*a*), and left humerus USNM 186944 (*b*), of *Callophoca obscura* from Antwerp, Belgium, and the Lee Creek Mine, Aurora, North Carolina, respectively; uncatalogued left humerus (*c*) and left humerus USNM 187228 (*d*) of *Homiphoca* sp. from Langebaanweg, South Africa (*c*), and the Lee Creek Mine, Aurora, North Carolina (*d*); left holotype humerus MSNUP I-13993 (*e*) of *Pliophoca etrusca* from Tuscany, Italy; left holotype humerus USNM 181419 (*f*) of *Auroraphoca atlantica* from Lee Creek Mine, Aurora, North Carolina; and left holotype humerus USNM 639750 (*g*) of *Virginiaphoca magurai* dredged from the Nottoway River west of Franklin, Virginia. (*b*) and (*d*) have been illustrated by Koretsky & Ray [4]. Scale bar equals 10 cm. Image courtesy for *Pl. etrusca*: G Bianucci.

**Figure 4.**
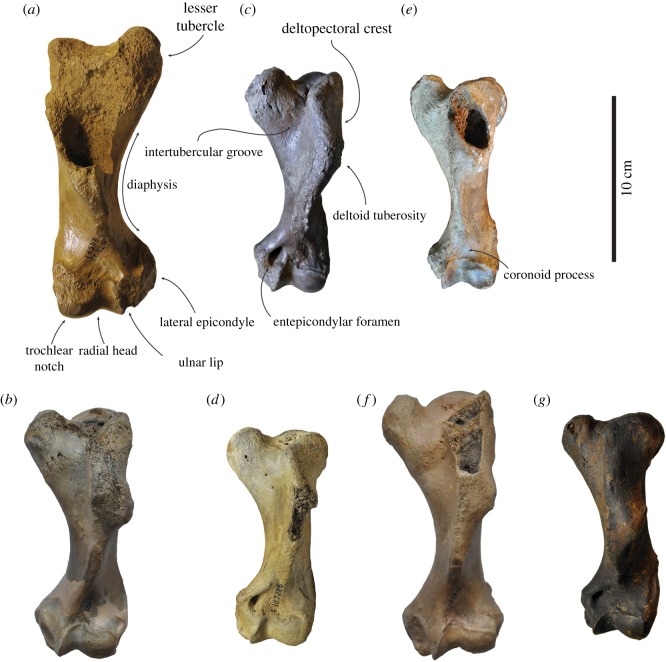
Comparison of humeri in anterior view. Right lectotype humerus IRSNB 1156-M177 (*a*), and left humerus USNM 186944 (*b*), of *Callophoca obscura* from Antwerp, Belgium, and the Lee Creek Mine, Aurora, North Carolina, respectively; uncatalogued left humerus (*c*) and left humerus USNM 187228 (*d*) of *Homiphoca* sp. from Langebaanweg, South Africa (*c*) and the Lee Creek Mine, Aurora, North Carolina (*d*); left holotype humerus MSNUP I-13993 (*e*) of *Pliophoca etrusca* from Tuscany, Italy; left holotype humerus USNM 181419 (*f*) of *Auroraphoca atlantica* from Lee Creek Mine, Aurora, North Carolina; and left holotype humerus USNM 639750 (*g*) of *Virginiaphoca magurai* dredged from the Nottoway River west of Franklin, Virginia. (*b*) and (*d*) have been illustrated by Koretsky & Ray [4]. Scale bar equals 10 cm. Image courtesy for *Pl. etrusca*: G Bianucci.

**Figure 5.**
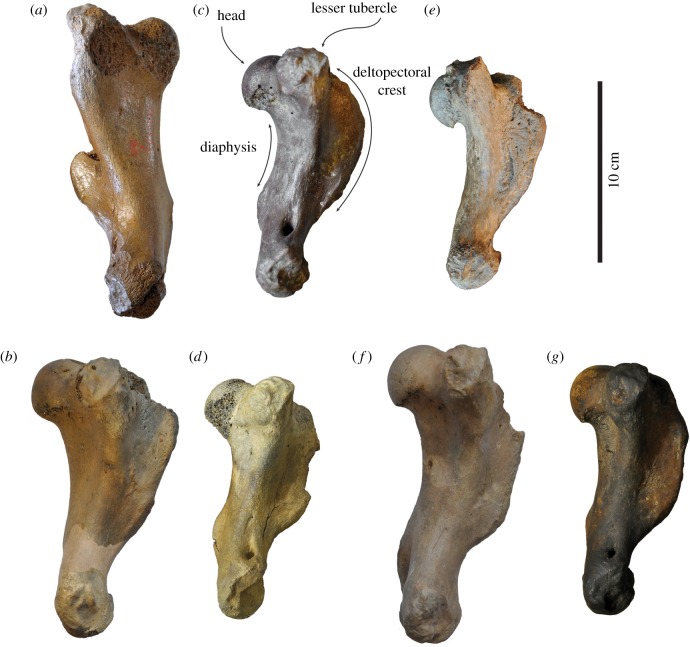
Comparison of humeri in medial view. Right lectotype humerus IRSNB 1156-M177 (*a*), and left humerus USNM 186944 (*b*), of *Callophoca obscura* from Antwerp, Belgium, and the Lee Creek Mine, Aurora, North Carolina, respectively; uncatalogued left humerus (*c*) and left humerus USNM 187228 (*d*) of *Homiphoca* sp. from Langebaanweg, South Africa (*c*), and the Lee Creek Mine, Aurora, North Carolina (*d*); left holotype humerus MSNUP I-13993 (*e*) of *Pliophoca etrusca* from Tuscany, Italy; left holotype humerus USNM 181419 (*f*) of *Auroraphoca atlantica* from Lee Creek Mine, Aurora, North Carolina; and left holotype humerus USNM 639750 (*g*) of *Virginiaphoca magurai* dredged from the Nottoway River west of Franklin, Virginia. (*b*) and (*d*) have been illustrated by Koretsky & Ray [4]. Scale bar equals 10 cm. Image courtesy for *Pl. etrusca*: G Bianucci.

**Figure 6.**
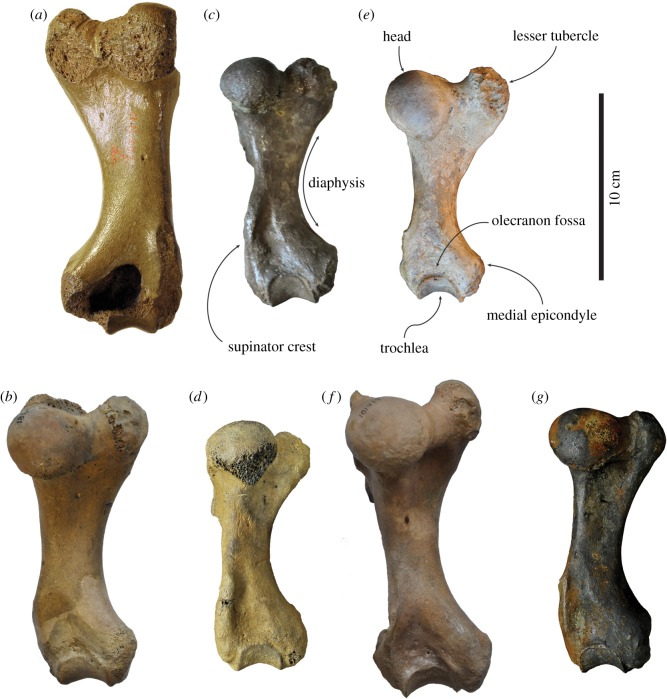
Comparison of humeri in posterior view. Right lectotype humerus IRSNB 1156-M177 (*a*), and left humerus USNM 186944 (*b*), of *Callophoca obscura* from Antwerp, Belgium, and the Lee Creek Mine, Aurora, North Carolina, respectively; uncatalogued left humerus (*c*) and left humerus USNM 187228 (*d*) of *Homiphoca* sp. from Langebaanweg, South Africa (*c*), and the Lee Creek Mine, Aurora, North Carolina (*d*); left holotype humerus MSNUP I-13993 (*e*) of *Pliophoca etrusca* from Tuscany, Italy; left holotype humerus USNM 181419 (*f*) of *Auroraphoca atlantica* from Lee Creek Mine, Aurora, North Carolina; and left holotype humerus USNM 639750 (*g*) of *Virginiaphoca magurai* dredged from the Nottoway River west of Franklin, Virginia. (*b*) and (*d*) have been illustrated by Koretsky & Ray [4]. Scale bar equals 10 cm. Image courtesy for *Pl. etrusca*: G Bianucci.

***Callophoca obscura*** Van Beneden, 1876

(figures 3*a*,*b*, 4*a,b*, 5*a,b* and 6*a,b*)

**Synonyms.** *Mesotaria ambigua* Van Beneden, 1876; *Phoca* (*Callophoca*) *obscura* Trouessart, 1897.

**Emended diagnosis.** Large monachine seal, comparable in size to *Hydrurga leptonyx* (2.8–3.6 m). *Callophoca obscura* differs from other extinct Monachinae by the presence of a lesser tubercle that exceeds the proximal level of the humeral head (except *Virginiaphoca magurai*, newly described taxon in this study), and a deltopectoral crest that is both broad and subtriangular in lateral view. Differs from all extant Monachinae by the smaller anterior projection of the deltopectoral crest and less curving distal termination of the deltopectoral crest. Additionally differs from *H. leptonyx* and *Leptonychotes weddellii* by the presence of a bicipital groove.

**Lectotype. IRSNB 1198-M203**, partial right humerus, ‘Scaldisian’ from the ‘third section’ at Deurne, Antwerp, Belgium. Ilustrated by Van Beneden ([3]: pl. 11, figs 1–4). Lectotype selected by Koretsky & Ray [4].

**Type locality and age.** Third section at Deurne, Antwerp, Belgium. The ‘third section’ follows Van Beneden's discretization of the nineteenth-century fortification constructions around the city of Antwerp, with the third section at Deurne being located southwest to the Deurne district of Antwerp ([8]: fig. 1) [3,38]. However, it should be noted that this type locality is derived from the original labels associated with the specimen. In his original publications Van Beneden [2,3] did not discuss the geographical provenance of individual specimens of the original fossil record of *C. obscura*.

V. Beneden (1877, unpublished data) assigned the specimen IRSNB 1198-M203 to the ‘Scaldisien’ (Scaldisian). However, as mentioned above, the Scaldisian is currently considered an obsolete term and the name is no longer used [25]. In addition, many of the apparently Scaldisian phocid remains show signs of reworking and for *Nanophoca vitulinoides* it has already been shown that the actual fossil record is older than the Pliocene range that is commonly accepted for Scaldisian phocids ([13] contra [4])*.* Indeed, dinoflagellate cyst biostratigraphy of sediment sample associated with *C. obscura* (specimen IRSNB 1156-M177) suggests a Messinian age for the sediment sample, thus most likely adjusting the known stratigraphic age for the lectotype of *C. obscura* from the early Pliocene to the late Miocene (electronic supplementary material).

**Other referred specimens. IRSNB 1116-M188**. Partial right juvenile humerus missing the proximal epiphysis, from the ‘Scaldisian’ of the ‘third section’ at Deurne, Antwerp, Belgium (originally illustrated as *Paleophoca nystii* by Van Beneden ([3]: pl. 10, figs 10–13). **IRSNB 1156-M177***.* Partial right humerus, from the ‘Scaldisian’ of the ‘third section’ at Borgerhout, Antwerp, Belgium (illustrated as *Mesotaria ambigua* by Van Beneden ([3]: pl. 9, figs 9–11). **IRSNB VERT-17172-301b***.* Complete left humerus, from an unknown stratigraphic position at Deurne, Antwerp, Belgium. USNM 186944. Incomplete left humerus, from the early Pliocene Yorktown Formation at the Lee Creek Mine, Aurora, Beaufort County, North Carolina (illustrated by Koretsky & Ray [4]: figs 24E, 25E, 26E, 27E).

**Comments.** Historically, *Callophoca obscura* is a very well-known phocid from the southern margin of the North Sea Basin (i.e. Belgium) and the Lee Creek Mine from North Carolina, USA, yielding well over one thousand isolated bones of the North Atlantic Ocean, including a rare partial cranium from North America (USNM 475486) (e.g. [2–4]; C.M. Peredo and L. Dewaele 2017, personal observation). As a result, *C. obscura* has been considered in many phylogenetic analyses of Phocidae, and Monachinae in particular (e.g. [12,16,39–42]). However, all specimens that have diagnostic value (i.e. cranium, humeri, femora) have been found isolated, and only humeri can be compared with the lectotype humerus. All other types of bones have only tentatively been assigned to *C. obscura* in the past on the basis of the size of the specimens and the relative abundances in collections (e.g. [3,4]). Although it is likely that most of these specimens indeed belong to *C. obscura*, we invoke a conservative approach and reassign all non-humerus specimens from *C. obscura* as Monachinae indet. An individual reassessment of all specimens is beyond the scope of this study and we limit our research to the humeri of the species.

Koretsky & Ray [4] synonymized *Callophoca obscura* and *Mesotaria ambigua* because they observed no anatomical differences other than size, arguing that size difference alone represents sexual dimorphism: the smaller *C. obscura* are proposed to represent females whereas the larger *M. ambigua* would represent males of the same species. Apart from *M. ambigua*, Koretsky & Ray [4] also considered *Paleophoca nystii* Van Beneden, 1876 (and alternative spellings, including *Monachus* (*Pristiphoca*) *nystii* Trouessart, 1897; *Palaeophoca nystii* Hendey, 1972; *Paläophoca nystii* Toula 1897; *Palaeophoca nysti* Allen, 1880; *Paloeophoca nysti* Allen, 1880; *Phoca Nystii* Gervais, 1872; *Poleophoca nystii* Van Beneden, 1876) a junior synonym to *C. obscura*. Many of the postcranial specimens that have formerly been assigned to *Pa. nystii* are notably similar to the postcranial bones of *C. obscura*. However, the holotype of *Pa. nystii* is an isolated tooth [4,43] that has later been reassigned to the odontocete cetacean *Scaldicetus grandis* (du Bus, 1872) [4]. Later, Bianucci & Landini [44] questioned the validity of *S. grandis*, restricting the taxon to its non-diagnostic holotype. Therefore, *Pa. nystii* cannot technically be considered a synonym to either *C. obscura* or *S. grandis*.

Considering the material from the east coast of North America, most of the humeri which have been assigned to *Pliophoca etrusca* by Koretsky & Ray [4] seem to belong to *C. obscura*. In addition, Berta *et al*. [12] noted marked differences between collections from the Eastern and Western Atlantic, considering *C. obscura* a nomen dubium because the taxon is based on isolated specimens, which they deemed dubious for reliable taxonomy.

**Description and comparison**

*Humerus*. Because the lectotype of *Callophoca obscura* is an isolated partial humerus (IRSNB 1198-M203), other humeri are the only bones that can unambiguously be compared and assigned to *C. obscura* (figures 3*a,b*, 4*a,b*, 5*a,b* and 6*a,b*). Four isolated humeri from Belgium and a few tens of specimens from the east coast of North America can be assigned to the taxon. The humeral head is slightly compressed proximodistally, as stated by Koretsky & Ray [4], and the head faces proximoposteriorly, while it projects more posteriorly in *Pliophoca etrusca*. The humeral neck is poorly developed in *C. obscura*, while it is much more prominent in the holotype of *Pl. etrusca* and strongly overhangs the diaphysis posteriorly. The lesser tubercle reaches proximal to the level of the humeral head. This strongly resembles extant Phocidae (except *Monachus*) which all have a strongly developed lesser tubercle, reaching higher than the level of the humeral head, while other late Neogene Monachinae (e.g. *Acrophoca longirostris*, *Homiphoca capensis*, *Piscophoca pacifica* and *Pl. etrusca*) all tend to have a lesser tubercle that approximately reaches the same level as the humeral head, or distal to it (e.g. [12,22,23,32,34]). The greater tubercle is also well-developed, reaching the proximal level of the humeral head. A similar condition has been observed in the Neogene Monachinae *Ac. longirostris*, *Auroraphoca atlantica* (newly described taxon in this study), *H. capensis* and *Pi. pacifica*, in which the greater tubercle reaches the same proximal level as the humeral head, or exceeds it. This contrasts with other Monachinae in which the greater tubercle does not reach the proximal level of the humeral head [22–24].

The deltopectoral crest is poorly preserved in the lectotype humerus IRSNB 1198-M203 of *C. obscura*, but it is preserved in North American specimens. It is separated from the lesser tubercle by a wide and deep intertubercular groove. In the intertubercular groove, a distinct transverse bicipital bar is present, as in most Monachinae, except the extant *Hydrurga leptonyx*, *Leptonychotes weddellii* and the extinct *Ac. longirostris*, *Homiphoca* sp., and the newly described *Virginiaphoca magurai* [22,32]. The deltopectoral crest of *C. obscura* differs from that in other Monachinae in that it is both broad (contra other extinct Monachinae) and rounded triangular (contra extant Monachinae) in lateral view ([24]: fig. 4). As in other Monachinae, the deltopectoral crest of *C. obscura* is rounded and distally terminates smoothly just proximal to the coronoid fossa (compare [24]: fig. 4). In lateral view, the deltopectoral crest is widest proximally. The deltoid tuberosity is strongly pronounced on the lateral side of the deltopectoral crest and located just proximal to the middle of the bone. In anterior view, the deltopectoral crest is slightly curving laterally and slightly offset laterally, as in *Homiphoca*, while it is straight in *Pl. etrusca*. In lateral view, the deltopectoral crest of *C. obscura* is uniquely broad yet subtriangular; and diaphysis has only a minor curvature. Extant Monachinae all have a diaphysis with strong curvature, while many extinct Monachinae retain a straighter diaphysis, such as *Ac. longirostris*, *Pi. pacifica* and *Pl. etrusca*. Yet, a number of extinct Monachinae, including *Homiphoca* and *Properiptychus argentinus* have a rather strongly curving diaphysis [12,15,22,23,32,45].

At the distal extremity of the humerus of *C. obscura*, the coronoid fossa on the anterior side is weakly developed, as is the olecranon fossa on the posterior side. The supinator crest on the lateral epicondyle is only little developed, which is a typical monachine trait. The medial epicondyle is better developed and appears as a rounded obtuse triangle in anterior view. *Callophoca obscura* lacks an entepicondylar foramen, similar to all other Monachinae, except *Homiphoca* and *V. magurai* nov. gen. et nov. sp. At the trochlear notch, the anterior margin of the radial head strongly slopes distolaterally, as in *Homiphoca*. In *Pliophoca etrusca*, this margin is more proximally convex.

***Homiphoca*** Muizon and Hendey, 1980

**Type and only included species.** *Homiphoca capensis* (Hendey and Repenning, 1972)

**Diagnosis.** As for the species.

*Homiphoca capensis* (Hendey and Repenning, 1972)

(figures 3*c*,*d*, 4*c,d*, 5*c,d*, 6*c,d*)

**Synonyms.** *Prionodelpis capensis* Hendey and Repenning, 1972

**Diagnosis.** As presented by Muizon & Hendey [32, pp. 94–96]: ‘A monachine phocid with a skull superficially similar to that of *Monachus*. It differs from *Monachus* in having a relatively large rostrum, which is wide posteriorly and narrow anteriorly. As in *Monachus*, but unlike Lobodontini, the premaxillae terminate against the nasals, where they are anteroposteriorly elongated. The premaxillae have prominent tuberosities anteriorly. The ascending process of the maxilla is relatively high as in Lobodontini and, viewed anteriorly, is not strongly recurved medially as in *Monachus*. Dental formula: 2.1.4.1/2.1.4.1. The premolars are morphologically similar to those of *Monachus* and unlike those of Lobodontini. They differ from those of *Monachus* in being lower crowned, relatively narrow and in having a pronounced posterolingual expansion of the cingulum. The accessory cusps on the premolars are small but distinct, while the M_1_ usually lacks such cusps and is distinct in having a strongly recurved and sharp, pointed principal cusp. The M_1_ is the largest of the cheek teeth, with the principal cusp slanted posteriorly, and often with a small accessory cusp low on the long anterior keel of the principal cusp. The interorbital region is broad and tapers posteriorly as in crabeater seal *Lobodon carcinophaga*, but unlike all other monachines. In the auditory region the tympanic bulla covers the petrosal, while the mastoid forms a lip overlapping the posterior border of the bulla.’

**Holotype. SAM-PQ-L15695**, incomplete and partially restored cranium, including left C and P4, and right P3, (illustrated as *Prionodelphis capensis* by Hendey & Repenning, ([45]: fig. 1); and as *Homiphoca capensis* by Govender *et al*. ([34]: fig. 2E, F) and Govender ([46]: figs 4, 8A]).

**Type locality and age.** ‘E’ Quarry, Langebaanweg, Cape Province, South Africa. Originally from ‘bed 2’ [45], reassigned to the Langeberg quartzose sand and Muishond Fontein peletal phosphorite members of the Varswater Formation, with an age of 5.15 ± 0.1 Ma (Zanclean, early Pliocene) [33].

**Comments.** Historically, *Homiphoca capensis* was represented by more than 3000 specimens from the ‘E’ Quarry, Langebaanweg, South Africa [32,34], and a handful of isolated specimens from the Lee Creek Mine in North Carolina [4]. Recent research of the *Homiphoca* material of the ‘E’ Quarry by Govender *et al*. [34] and Govender [46] showed the existence of at least two different morphotypes of *Homiphoca* in the fossil record. Govender *et al*. [34] presented two morphotypes of the cranium, scapula, humerus, radius, ulna, innominate, femur, and tibia and fibula; with morphological variation apparently exceeding that of intraspecific variation based on extrapolation of extant Monachinae. Later, Govender [46] performed a detailed analysis of the known crania of *Homiphoca*, confirming the presence of two (and possibly three) different morphotypes of *Homiphoca* crania.

Moreover, there is considerable disagreement on the possible existence of a yet-undescribed species of *Homiphoca*. Some researchers suggest that the observed morphological differences between the different morphotypes exceeds intraspecific variation [34,46], while others rather invoke profound sexual dimorphism and consider it unlikely for multiple closely related species to occupy the same ecological niche in the same environment and at the same time (C. de Muizon 2017, personal communication; L. Dewaele 2017, personal observation). Because the fossil record of *Homiphoca* consists of isolated crania, mandibles and postcranial bones, it is impossible to relate the holotype cranium to any of the isolated postcranial specimens, or to compare the different isolated postcranial bones reciprocally. Therefore, it is impossible to ascertain the correlation between the different morphotypes to different types of bones. Consequently, we judge it appropriate to consider the entire fossil record of *Homiphoca* with extreme care and only consider the holotype cranium SAM-PQ-L15695 to belong to *H. capensis* unquestionably. Following the reasoning for *Callophoca obscura*, all postcranial specimens of *H. capensis* should be considered as indeterminate monachine. However, the stratigraphy of *Homiphoca* from South Africa differs from *C. obscura* from the North Atlantic. Virtually all cranial and postcranial specimens attributed to *H. capensis* come from the LQSM and MPPM levels of the Varswater Formation in South Africa [32–34,46]. These levels have a very limited temporal range, and are virtually bonebeds. Hence, it can be argued as to what extent multiple closely related taxa can occupy the same ecological niche in the same area and at the same time. Furthermore, the high number of specimens found at Langebaanweg, the only known South African locality of *Homiphoca* renders it very likely that at least one of both morphotypes of humeri belongs to *H. capensis*. Therefore, it is safe to assume that all isolated seal specimens, which can be grouped into two morphotypes, may actually represent one sexually dimorphic taxon. But, pending the discovery of more complete articulated skeletons of *H. capensis*, we judge it most appropriate to consider specimens *Homiphoca* sp., rather than *H. capensis* or Monachinae indet. The stratigraphic range of *C. obscura*, on the other hand, is completely different in that the stratigraphic range of *C. obscura* from Belgium is poorly delineated and that the Yorktown Formation in North America had been deposited over a longer time span [30,47] with no clear fossiliferous horizon and most fossils recovered from spoil piles with no detailed stratigraphic context other than the formation [48]. The ‘*H. capensis*’ specimens of the Lee Creek Mine in North Carolina should conservatively be considered *Homiphoca* sp. as well (contra Koretsky & Ray [4]). A reassessment of the *Homiphoca* humeri from Koretsky & Ray [4] is presented below.

***Homiphoca*** sp.

**Locality.** Lee Creek Mine, Aurora, Beaufort County, North Carolina, USA.

**Stratigraphy and age.** Yorktown Formation. Zanclean, lower Pliocene (see above; [30])

**Referred specimen. USNM 187228**, subcomplete right humerus, from the early Pliocene Yorktown Formation at the Lee Creek Mine, Aurora, Beaufort County, North Carolina (illustrated by Koretsky & Ray [4]: fig. 50E–H).

**Comments.** The holotype specimen of *Homiphoca capensis*, the sole species currently defined in the genus *Homiphoca*, is a partial cranium that has been found isolated [32,45]. Recent research by Govender *et al*. [34] and Govender [46] showed that virtually all cranial and postcranial specimens attributed to *H. capensis* have been found isolated and that at least two different morphotypes exist, hypothesizing the presence of at least a second species of *Homiphoca*. However, the presence of two closely related taxa in the same locality and occupying the same ecological niche is questionable, as noted above.

**Description and comparison**

*Humerus*. Koretsky & Ray [4] assigned two humeri from the Yorktown Formation at the Lee Creek Mine to *Homiphoca capensis*: USNM 187228 and USNM 214550 (figures 3*c,d*, 4*c,d*, 5*c,d*, 6*c,d*). However, the whereabouts of the latter specimen are unknown and the current study only focuses on specimen USNM 187228. The humeral head of *Homiphoca* specimen USNM 187228 from the east coast of North America faces proximoposteriorly, similar to *Callophoca obscura* and *Homiphoca* from South Africa. The neck is well developed and it is morphologically similar to that of *Homiphoca* from South Africa, but unlike that of *C. obscura* in which it is less developed, or *Pliophoca etrusca* in which it is also well developed but more strongly overhanging the diaphysis. The lesser tubercle of *Homiphoca* from North America reaches the proximal level of the humeral head, as in *C. obscura*, *Homiphoca* from South Africa, and *Virginiaphoca magurai* nov. gen. et nov. sp. (see below) from the Nottoway River, but unlike *Pl. etrusca* and *Auroraphoca atlantica* nov. gen. et nov. sp. (see below) previously considered *Pl. etrusca* from North America, which has a lesser tubercle that exceeds the level of the humeral head. A reduced lesser tubercle is considered a plesiomorphic trait among pinnipeds [22,49]. The lesser tubercle diverts strongly off the diaphysis. The greater tubercle does not reach the proximal level of the humeral head, which is considered a derived character in Phocidae, but appears to be present in all extant Phocidae and only some extinct Phocidae (see, e.g. Dewaele *et al*. [11,13]).

The deltopectoral crest is moderately well preserved in USNM 187228. It is separated from the lesser tubercle by a wide intertubercular groove. In the intertubercular groove, there is no transverse bicipital bar present. As mentioned above, such a bicipital bar is present in most Monachinae, except the extant *Hydrurga leptonyx*, *Leptonychotes weddellii* and the extinct *Acrophoca longirostris*, *Homiphoca* sp. from South Africa and *V. magurai* [22]. The deltopectoral crest of USNM 187228 is rounded and terminates distally proximal to the coronoid fossa. On the lateral side of the deltopectoral crest, the deltoid tuberosity overhangs the diaphysis, along the entire length of the tuberosity. This condition varies among Neogene Monachinae from the North Atlantic. In anterior view, the deltopectoral crest is slightly curving laterally and slightly offset laterally, as in *C. obscura* and *Homiphoca* from South Africa, while it is straight in *Pliophoca etrusca*. In lateral view, the diaphysis has a strong curvature, as in *Homiphoca* from South Africa and extant Monachinae. Many other extinct Monachinae retain a straighter diaphysis, such as *Ac. longirostris*, *C. obscura*, *Piscophoca pacifica* and *Pl. etrusca* [12,22].

At the distal extremity of the humerus of USNM 187228, the coronoid fossa on the anterior side is weakly developed, as is the olecranon fossa on the posterior side. The supinator crest on the lateral epicondyle is better developed than in other Monachinae, except *Homiphoca* from South Africa, and hence, also better than in *C. obscura* and *Pl. etrusca*. The medial epicondyle bears an entepicondylar foramen, which is a symplesiomorphic trait shared with Phocinae and other Pinnipedia, except Monachinae. Among Monachinae, the presence of an entepicondylar foramen is unique to *Homiphoca* and *V. magurai* nov. gen. et nov. sp. (USNM 639750, see below). *Monotherium* also has an entepicondylar foramen and has been considered a monachine [12,22], but the humeri of *Monotherium aberratum* and *Monotherium affine* Van Beneden, 1876 are considered to be phocine in a recent study by Dewaele *et al*. [50]. Overall morphology and the description of the *Homiphoca* specimens from the Lee Creek Mine, show noticeable similarities with the *Homiphoca* humeri from South Africa. The study of Govender *et al*. [34] shows relatively little difference between the two humerus morphotypes of *Homiphoca* from South Africa. Two of the most prominent differences observed are the degree of development and curvature of the deltopectoral crest and the shape of the deltoid tuberosity. The deltoid tuberosity is very incompletely preserved in specimen USNM 187228. The shape of the deltopectoral crest of USNM 187228 corresponds to that of morphotype I from South Africa, in which the deltopectoral crest ‘does not follow the curve of the bone’ [34, p. 142]. Overall, the humerus USNM 187228 is strongly similar to that of morphotype I of *Homiphoca* from South Africa, and differences are negligible. Therefore, it is indeed safe to consider *Homiphoca* sp. to be present in the Yorktown Formation of the east coast of North America.

***Pliophoca*** Tavani, 1941

**Type and only included species**. *Pliophoca etrusca* Tavani, 1941

**Diagnosis.** As for the species.

***Pliophoca etrusca*** Tavani, 1941

(figures 3*e*, 4*e*, 5*e*, 6*e*)

**Emended diagnosis.** We retain the holotype specimen, described by Tavani [15], and redescribed by Berta *et al*. [12] as the sole unquestionable specimen of *Pliophoca etrusca*. We only emend the part of the humerus in the diagnosis presented by Berta *et al*. [12] and exclude *Leptophoca* True, 1906 from the list of stem monachines, following recent recent phylogenetic analysis of the taxon by Dewaele *et al*. [11,13]: *Pliophoca* is distinguished from *Monachus* by medial tuberosity on premaxillary reduced or absent, lateral extension on premaxillary present, upper incisors transversely compressed, femur epiphyses in which the distal epiphysis is wider than the proximal epiphysis, greater trochanter of femur higher than the head, and calcaneum slightly longer than the astragalus. *Pliophoca* is distinguished from stem monachines (i.e. *Acrophoca*, ‘*Callophoca*’, *Homiphoca* and *Piscophoca*) by the following derived characters: humeral head strongly overhanging the humeral neck, supinator ridge on humerus absent or poorly developed and metacarpal I longer than metacarpal II. *Pliophoca* is distinguished from *Mirounga* and lobodontines in retention of the following primitive characters: medial tuberosity on the premaxillary reduced or absent, mastoid lip does not cover the external cochlear foramen, procumbent upper incisors absent and intercondylar region of femur narrow and deep.

**Holotype. MSNUP I-13993**, partial skeleton including the cranium (rostrum and fragmentary left half of the cranium); fragmentary cervical, thoracic, lumbar, sacral and caudal vertebrae; partial fore flippers (including a subcomplete left humerus, missing part of the deltopectoral crest) and partial hind flippers (illustrated as *Pliophoca etrusca* by Tavani ([15]: figs 1, 2, 10, 13, 20, 24) and Berta *et al*. ([12]: figs 2, 3A–C, 3F, 4–10).

**Type locality and age.** Casa Nuova, Orciano Pisano, Tuscany, Italy. Piacenzian (late Pliocene) layer of the Argille Azzurre Formation, dated at 3.19–2.82 Ma [36].

**Comments.** The holotype specimen MSNUP I-13993 is the only known specimen that can be attributed to the species *Pliophoca etrusca* [12]. However, this holotype is relatively complete, including a partial cranium, limb bones and vertebral bodies [12,15,35], making it one of the most completely known phocid fossils. Originally, Tavani [35] assigned additional material to the species: A fragment of a mandible from Orciano Pisano and specimens attributed to multiple individuals, including the left and right mandibles of a single individual and multiple other cranial, axial and appendicular bones from near Saline di Volterra, are housed at the Museo di Geologia e Paleontologia in Florence and were referred to *Pl. etrusca* by Tavani [35]. However, Berta *et al*. ([12]: e889144-16) approached the material more conservatively and only retained the holotype specimen as *Pl. etrusca*, arguing about the additional specimens that ‘most of it is from a different locality than the holotype and it consists, in part, of some noncomparable elements to the holotype (e.g. mandible), we feel that it is more appropriately referred to *Pliophoca* cf. *Pl. etrusca*’. In the current study, we encourage a more conservative approach in phocid palaeontology, advocating against the widespread practice of assigning isolated incomparable specimens to one or another taxon. Therefore, we support Berta *et al*. [12] in considering that it is not appropriate to consider the very partial specimens from the Museo di Geologia e Paleontologia in Florence as *Pl. etrusca*. In their review of Pliocene Phocidae from the east coast of North America, Koretsky & Ray [4] presented 171 specimens of *Pl. etrusca* from different localities in North Carolina and Florida: mostly isolated bones, but also including one associated scapula, humerus, radius and ulna (USNM 250290), and one associated humerus and femur (USNM 374222). Furthermore, this collection includes 16 maxillae, 8 isolated maxillary teeth, 44 mandibles, 18 isolated mandibular teeth, 35 humeri, 24 radii and 17 femora, based on the study by Koretsky & Ray [4]. However, these specimens show little similarities with the holotype from Italy, and are all reassigned to other taxa (see below) or as indeterminate Monachinae. This has also been postulated by Berta *et al*. [12], considering Koretsky & Ray's [4] allegedly North American specimens of *Pl. etrusca* as ‘*Pliophoca* aff. ‘*Pl. etrusca*’ USNM’.

Genus ***Auroraphoca*** nov. gen.

**LSID.** urn:lsid:zoobank.org:act:32C1A028-255C-4E3C-9F29-11A3CE7E5802

**Type and only included species.** *Auroraphoca atlantica* nov. gen. et nov. sp.

**Etymology.** From the toponym ‘*Aurora*’ and the Greek noun ‘*phoke*’. ‘*Aurora*’ is the town in Beaufort County, North Carolina where the Lee Creek Mine is located. The Yorktown Formation in the Lee Creek Mine is one of the most prolific fossil seal localities in the Northern Hemisphere [4] and is also the locality where the holotype of *Auroraphoca atlantica* was discovered. ‘*Phoké*’ means ‘seal’.

**Diagnosis.** As for the species.

**Figure 7.**
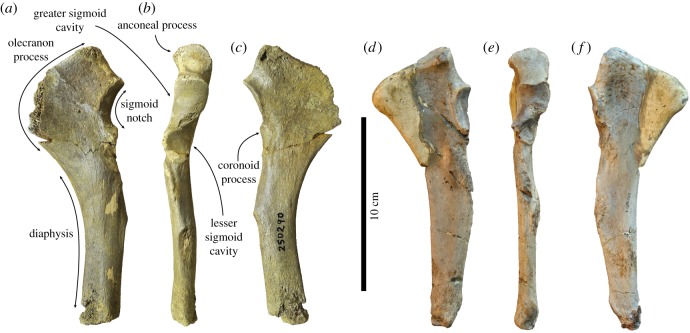
Left ulna USNM 250290 of *Auroraphoca atlantica* in (*a*) medial, (*b*) anterior, and (*c*) lateral view, formerly considered to represent *Pliophoca etrusca* from the Lee Creek Mine, Aurora, Beaufort County, North Carolina, USA [4]. This specimen is considerably different from the holotype left ulna MSNUP I-13993 from *Pl. etrusca* of Italy, in (*d*) medial, (*e*) anterior and (*f*) lateral view. Note the incompleteness of the olecranon process in both specimens, which has been reconstructed in specimen MSNUP I-13993 (*d,f*). Scale bar equals 10 cm. Image courtesy for *Pl. etrusca*: G Bianucci.

***Auroraphoca atlantica* nov. gen. et nov. sp.**

(figures 3*f*, 4*f*, 5*f*, 6*f*, 7*a–c*)

**LSID.** urn:lsid:zoobank.org:act:1C376335-3827-4B67-BFE7-DE4A6B5CF443

**Diagnosis.** *Auroraphoca atlantica* is a medium-sized phocid, comparable in size to the extant *Lobodon carcinophaga*, different from other extant and other extinct Monachinae by the abrupt distal termination of the deltopectoral crest, and the presence of a reduced and distally located epicondylar crest. It differs from all extinct Monachinae (except *C. obscura*) by the strong development of the lesser tubercle, exceeding the proximal level of the humeral head, and from all extinct Monachinae (except *Pliophoca etrusca*) by the weak development of the anconeal process on the ulna.

**Etymology.** The specific name ‘*atlantica*’ refers to the Atlantic Ocean. The holotype specimen of *Auroraphoca atlantica* was recovered from the Yorktown Formation, deposited at the east coast of North America during the Pliocene.

**Holotype. USNM 181419**, incomplete left humerus, from the early Pliocene Yorktown Formation at the Lee Creek Mine, Aurora, Beaufort County, North Carolina (illustrated as *Pliophoca etrusca* by Koretsky & Ray ([4]: figs 24E, 25E, 26E, 27E).

**Paratype. USNM 250290**, articulated proximal portion of a left scapula, partial left humerus and left ulna, from the early Pliocene Yorktown Formation at the Lee Creek Mine, Aurora, Beaufort County, North Carolina (illustrated as *Pliophoca etrusca* by Koretsky & Ray [4] figs 44, 47).

**Type locality.** Lee Creek Mine, Aurora, Beaufort County, North Carolina, USA.

**Type horizon.** Yorktown Formation. Zanclean, lower Pliocene, based on foraminifer biostratigraphy [30].

**Description and comparison**

*Scapula*—One very incomplete scapula can be assigned to *Auroraphoca atlantica*. Of this bone, from specimen USNM 250290, only the proximal portion is preserved, but its identification as *Au. atlantica* is based on its articulation with a humerus that can be identified as *Au. atlantica*. The specimen is not described here because it is too incompletely preserved, rendering the significance of a description futile.

*Humerus*—Humeri USNM 181419 and USNM 250290 differ morphologically from both *Callophocaobscura* and *Pliophoca etrusca*, although Koretsky & Ray [4] formerly assigned both specimens to *Pl. etrusca* (figures 3*f*, 4*f*, 5*f*, 6*f*). Humerus USNM 181419 is nearly complete, missing only the anteroproximal portion of the deltopectoral crest. Humerus USNM 250290 is associated with a partial scapula and an ulna. This specimen is only partially preserved and is clearly not an adult, as can be judged from the clearly visible suture between the proximal epiphysis and the diaphysis. The humerus of *Auroraphoca atlantica* is overall relatively slender compared with other contemporaneous monachine humeri [4,12,15,32] (table 1). The humeral head is rounded, hemispherical and much smaller than in the holotype humerus of *Pl. etrusca*. The humeral head appears negligibly smaller than in other contemporaneous Monachinae from the North Atlantic and *Homiphoca* from South Africa. The greater tubercle extends proximally to the humeral head, and the lesser tubercle reaches proximal to both the humeral head and the greater trochanter. Among Monachinae, and Phocinae in general, the development of the lesser and great tubercles of the humerus show evolutionary trends [11,13,22]. Among Phocidae, the development of the greater and lesser trochanter shows opposite evolutionary trends, with the greater trochanter usually being well developed in extant Phocidae (except *Monachus monachus)*, and less well developed in extinct Phocidae (except *Callophoca obscura* and *Pl. etrusca*) ([11,13]; this study). Dewaele *et al*. [11,13] and Muizon [22] noted the reverse for the development of the lesser tubercle of the humerus in Phocidae. The strong development of the lesser tubercle in the humerus of *Au. atlantica* corresponds more with extant Phocidae and *C. obscura* and *Pl. etrusca* than with most extinct Phocidae. A broad bicipital groove separates the lesser tubercle from the greater tubercle and the deltopectoral crest. In the intertubercular groove, there is a transverse bicipital bar. The deltopectoral crest extends for almost two-thirds along the anterior side of the diaphysis, terminating rather abruptly, distally. This abrupt distal termination separates the humerus of *Au. atlantica* from the humeri of extant and other extinct Monachinae, which all have a smooth and more gradual distal termination [49,51]. The deltoid tuberosity extends along the proximal two-thirds of the lateral side of the deltopectoral crest. This tuberosity overhangs the diaphysis laterally in two distinct places, anteriorly and posteriorly. This condition varies among different taxa of Monachinae from the Neogene of the North Atlantic. A similar condition to *Au. atlantica* is observed only in the newly described humerus USNM 639750 from *Virginiaphoca magurai* (see below). In anterior view, the deltopectoral crest of humerus USNM 181419 is slightly convex laterally, and the crest terminates above the coronoid process. The diaphysis of the humerus of *Au. atlantica* is slender compared with the humerus of other Monachinae from the Neogene of the North Atlantic. Proximally on the posterior surface of the diaphysis of the humerus there is a deep fossa serving as the attachment surface for the triceps brachii muscle. Among phocids, a similar condition has only been observed in *Monotherium aberratum*, *Monotherium affine* and *Piscophoca pacifica* ([22]; L. Dewaele 2017, personal observation).

In anterior view, the distal epiphysis of USNM 181419 is equally wide as the proximal epiphysis. This character is shared with *Homiphoca* and with the newly described humerus from the Nottoway River (see below). In *C. obscura* and *Pl. etrusca*, the proximal epiphysis is broader than the distal epiphysis. The medial epicondyle strongly reaches distally. The epicondylar crest is poorly developed, but forms a short and prominent protuberance distally, compared with the condition in other Monachinae, resulting in a strongly reduced attachment surface for multiple manual extensor muscles. This condition is unique in Phocidae. In extant Monachinae, *Pl. etrusca*, and *V. magurai* nov. gen. et nov. sp., the epicondylar crest is absent or poorly developed, while it is moderately well developed along the epicondyle in other extinct Monachinae [12,22]. There is no entepicondylar foramen, a synapomorphy among Monachinae. Among Phocidae, only the extinct monachines *Homiphoca* and *Monotherium*, and Phocinae have an entepicondylar foramen [2–4,22,32,45,49,51,52]. Correlated, the weak development of the epicondylar crest yields an apparently straighter humerus in lateral view than it does in *Homiphoca* or *V. magurai* nov. gen. et nov. sp. from the Nottoway River, but not as straight as in *C. obscura* or *Pl. etrusca*. The olecranon and coronoid fossae are strongly reduced, and the trochlea is small. The medial ulnar lip of the trochlea is well-developed, forming a sharp ridge, as in *Pl. etrusca* and *V. magurai*, but unlike *C. obscura* and *Homiphoca*.

***Ulna*—**Contrasting to other types of bone, Koretsky & Ray [4] assigned only one ulna from North America to *Pliophoca etrusca*: USNM 250290, which is associated with a scapula and a humerus. In the current study, this specimen is reassigned to *Auroraphoca atlantica* (figure 7*a–c*). Although the proximal epiphysis of the specimen is fused to the diaphysis, the absence of the distal epiphysis argues that this specimen belonged to a skeletally subadult seal [23], as had been argued by Koretsky & Ray [4]. Although incomplete, the total length of ulna USNM 250290 of *Au. atlantica* falls near the estimated total length of 170 mm for the ulna of *Homiphoca* [22]. USNM 250290 is only slightly longer than the holotype ulna MSNUP I-13993 of *Pl. etrusca*, and the holotype MNHN.F.ORN1 of *M. mauretanica*, but still within the range of natural intraspecific variation (L. Dewaele 2017, personal observation). The state of preservation of the olecranon process in both USNM 250290 and *Pl. etrusca* specimen MSNUP I-13993 limits comparative description of the process. However, it appears that the olecranon process in USNM 250290 is more strongly sloping than in *Homiphoca* and *Pl. etrusca*. The proximal portion of the ulna of *M. mauretanica* is also strongly sloping, with a strongly concave proximal margin of the olecranon process [19]. The anconeal process on the medial side of the proximal portion of the olecranon process is weakly developed, as in *Pl. etrusca*. This condition had been considered a monachine trait by Hendey & Repenning [45] (except *Piscophoca pacifica*). The sigmoid notch is strongly concave, contrasting to *Homiphoca*, *M. mauretanica* and *Pl. etrusca*. Partly related to this strong curvature of the sigmoid notch in lateral view, the coronoid process of USNM 250290 is more strongly pronounced than in *Homiphoca* and *Pl. etrusca*. The greater sigmoid cavity is saddle-shaped and simple (reversed) tear-drop shaped in USNM 250290, while it is much more sigmoidal in the *Pl. etrusca* holotype MSNUP I-13993, in which the distal extremity of this cavity strongly deflects medially; and the lesser sigmoid cavity is circular and flat, while it is slightly concave in *Pl. etrusca*. Overall, the sigmoid notch of ulna USNM 250290 resembles that of *M. mauretanica*. However, comparison involved illustrations of *M. mauretanica* and no direct observations. Moreover, the limited diagnostic value of ulna for Phocidae precludes comparing USNM 250290 with *M. mauretanica*.

The diaphysis of the ulna USNM 250290 is robust and strongly curved. In lateral view, the diaphysis is thicker than in *Homiphoca*, *M. mauretanica* and *Pl. etrusca*. In anterior view, the diaphysis is approximately equal to the proximal portion of the coronoid process, in USNM 250290 and *Homiphoca*, while the proximal portion of the coronoid process is wider than the diaphysis in *Pl. etrusca*, and while the diaphysis is wider than the proximal portion of the coronoid process in *M. mauretanica*. In lateral view, the posterior margin of the diaphysis is strongly curving in USNM 250290, as in *Homiphoca* and *M. mauretanica*, but contrasting to *Pl. etrusca*, in which this margin is relatively straight.

Genus ***Virginiaphoca*** nov. gen.

**LSID.** urn:lsid:zoobank.org:act:C5C2FBDF-F501-4253-BAE1-F550BB5B7F4

**Type and only included species.** *Virginiaphoca magurai* nov. gen. et nov. sp.

**Etymology.** From the toponym ‘*Virginia*’ and the Greek noun ‘*phoke*’. ‘*Virginia*’ refers to the state of Virginia (United States of America), alluding to the locality of the holotype specimen being dredged off the riverbed of the Nottoway River near Franklin, Virginia. ‘*Phoké*’ means ‘seal’.

**Diagnosis.** As for the species.

**Type locality and age**. As for the species.

***Virginiaphoca magurai* nov. gen. et nov. sp.**

(figures 3*g*, 4*g*, 5*g*, 6*g*)

**LSID.** urn:lsid:zoobank.org:act:8035E57E-F9D4-40B3-9771-AA4EF0DC8EEC

**Diagnosis.** *Virginiaphoca magurai* is a medium-sized phocid, comparable in size to the extant phocine *Phoca vitulina* (1.5–1.9 m). *Virginiaphoca magurai* is typically monachine in that the deltopectoral crest terminates smoothly distally, and differs from extant and other extinct Monachinae by the following characters: strong proximodistal compression of the humeral head (except *Monachus* spp., *Ommatophoca rossii*), a very reduced overhanging humeral neck, and the presence of an entepicondylar foramen (also present in *H. capensis* and *Monotherium*). Differs additionally from *Homiphoca* sp. by the reduction of the epicondylar crest.

**Etymology.** The specific name ‘*magurai*’ is a tribute to Joseph ‘Joe’ Magura who discovered the holotype specimen.

**Holotype. USNM 639750**, subcomplete left humerus from the Nottoway River, west of Franklin, Virginia, USA.

**Type locality.** Nottoway River, west of Franklin, Virginia, USA. Approximate coordinates: 36°40′ N, 77°01′ W.

**Type horizon.** Cobham Bay Member of the Eastover Formation, or the Yorktown Formation. The Cobham Bay Member of the Eastover Formation is radiometrically dated to the late Miocene. The Yorktown Formation is dated to the Zanclean, lower Pliocene, based on foraminifer biostratigraphy and radiometric dating [30,31].

**Comments.** In addition to humerus USNM 639750, other phocid specimens, including two complete femora (USNM 639748 and 639749), two radii (USNM 639751 and 639752), a tibia (USNM 639753), and two metatarsals (USNM 639754 and 639755), as well as terrestrial mammal vertebra (USNM 639756) have been dredged off the Nottoway River bed. These show a similar state of preservation with respect to completeness of the bone and color. However, a detailed description of the other phocid specimens is beyond the scope of the present study.

**Description and comparison**

***Humerus***—The left humerus USNM 639750 of *Virginiaphoca magurai* is completely preserved. The humerus is short and robust (table 1; figures 3*g*, 4*g*, 5*g*, 6*g*). The humeral head is hemispherical and slightly compressed proximodistally. The degree of proximodistal compression is higher than in other monachine humeri from the Neogene of the North Atlantic. The neck is strongly reduced, compared with other Monachinae from the North Atlantic, giving the entire humerus a moderately straight appearance in lateral view: straighter than in *Homiphoca* sp. from North America and South Africa*,* similarly straight as humerus USNM 181419 of *Auroraphoca atlantica*, but not as straight as in *Callophoca obscura* or *Pliophoca etrusca*. Extant Phocidae and other extinct Phocidae all have a relatively more curved humeral diaphysis in lateral view. The lesser tubercle almost reaches the same proximal level as the head and does not divert strongly medially from the axis of the humerus. In living monachines, except the monk seals of the genus *Monachus*, the lesser tubercle is strongly developed and exceeds the level of the humeral head. This condition varies among extinct Monachinae, with, e.g. *C. obscura*, *Homiphoca* sp. and *Pl. etrusca* having a well-developed lesser tubercle, while *Acrophoca longirostris*, *Piscophoca pacifica* and *Properiptychus argentinus* from South America all have a relatively small lesser tubercle ([22,51]; this study). The bicipital groove is moderately deep and wide. In the intertubercular groove, there is no transverse bicipital bar. The greater tubercle reaches slightly lower than the humeral head, as in extant Phocidae [22]. However, a number extinct monachine taxa have a greater tubercle that exceeds the level of the humeral head, including *C. obscura* and *Homiphoca* sp. (this study). The deltopectoral crest is long, approximately two-thirds the length of the entire bone and reaching the level of the proximal portion of the medial epicondyle. In lateral view, the humerus USNM 639750 of *V. magurai* is semicircular. This condition is roughly intermediate between the less expanded deltopectoral crest of *Ac. longirostris*, morphotype II in *Homiphoca*, *Pl. etrusca* and *Pr. argentinus*; and the deltopectoral crest of *C. obscura*, morphotype I of *Homiphoca* and *Piscophoca pacifica* (see [12,15,22,23,34]). The deltoid rugosity on the lateral surface of the deltopectoral crest is proximodistally elongated, widest proximally and tapering distally, and slightly overhanging the lateral surface of the deltopectoral crest in two separate places. The latter condition is observed in the humerus of *Au. atlantica*, but otherwise this varies among extinct Monachinae. In anterior view, the deltopectoral crest of *V. magurai* does not deflect as much laterally as it does in other Neogene Monachinae from the North Atlantic, except *Pl. etrusca*, and largely remains within the anteroposterior plane through the axis of the humerus. Among Monachinae, the presence of an entepicondylar foramen is a feature unique to the genus *Homiphoca* [22,32,45,49]. An entepicondylar foramen is also known to exist in *Monotherium* [22]. However, a study contesting *Monotherium* as a monachine genus is in review. However, it is also present in USNM 639750. The distal portion of the humerus of *V. magurai* does not differ significantly from that of *Homiphoca*, with a broad and little-developed medial epicondyle. The epicondylar crest is little developed, as is common in all extant Monachinae, *Au. atlantica*, and *Pl. etrusca*, while it is moderately developed in other extinct Monachinae [12,22]. As Berta *et al*. [12] observed in *Pl. etrusca*, *V. magurai* has a narrow but well-marked attachment surface for the m. extensor carpi radialis on the posterolateral margin of the epicondylar crest. This attachment surface appears less strongly developed in *C. obscura* and *Homiphoca*. The coronoid fossa is rather shallow and the roughly oval olecranon fossa is moderately deep. The shared presence of an entepicondylar foramen in *V. magurai* and *Homiphoca* sp. and the absence of a transverse bicipital bar in the intertubercular groove may suggest a phylogenetic relationship between both taxa. However, the incompleteness of the fossil record of *V. magurai* inhibits a detailed phylogenetic analysis.

**Monachinae indet.**

**Locality.** Lee Creek Mine, Aurora, Beaufort County, North Carolina, USA.

**Stratigraphy and age.** Yorktown Formation. Zanclean, lower Pliocene [30].

**Referred specimens. USNM 187580.** Partial maxillae, from the early Pliocene Yorktown Formation at the Lee Creek Mine, Aurora, Beaufort County, North Carolina, USA (illustrated as *Pliophoca etrusca* by Koretsky & Ray [4]: fig. 41A, E). **USNM 181504.** Right radius, from the early Pliocene Yorktown Formation at the Lee Creek Mine, Aurora, Beaufort County, North Carolina (= *Pliophoca etrusca* Koretsky & Ray [4]). **USNM 307537.** Left radius, from the early Pliocene Yorktown Formation at the Lee Creek Mine, Aurora, Beaufort County, North Carolina (illustrated as *Pliophoca etrusca* by Koretsky & Ray [4]: fig. 46). **USNM 243686.** Left femur, from the early Pliocene Yorktown Formation at the Lee Creek Mine, Aurora, Beaufort County, North Carolina (= *Pliophoca etrusca* Koretsky & Ray [4]). **USNM 250293.** Left femur, from the early Pliocene Yorktown Formation at the Lee Creek Mine, Aurora, Beaufort County, North Carolina (= *Pliophoca etrusca* Koretsky & Ray [4]). **USNM 251209.** Left femur, from the early Pliocene Yorktown Formation at the Lee Creek Mine, Aurora, Beaufort County, North Carolina (= *Pliophoca etrusca* Koretsky & Ray [4]). **USNM 460248.** Right femur, from the early Pliocene Yorktown Formation at the Lee Creek Mine, Aurora, Beaufort County, North Carolina (= *Pliophoca etrusca* Koretsky & Ray [4]).

**Comments.** Fossil phocid specimens that Koretsky & Ray [4] assigned to *Pliophoca etrusca* from North America are reconsidered in this study (see below). Our reassessment shows that some specimens are incomparable with the holotype (and currently only definitely known) specimen of *Pl. etrusca*, such as is the case for mandibles and the mandibular dentition ([4] versus [12]). Specimens from the Neogene of North America that are comparable with the Italian holotype of *Pl. etrusca* show noticeable differences with the holotype, despite the designation by Koretsky & Ray [4] (see below). Consequently, given the overall incompleteness of the fossil record of Monachinae from the North Atlantic, most of these redescribed specimens are to be considered Monachinae indet.

In the current study, we argue that only nearly complete or complete humeri should be used in the designation of new monachine taxa, in the absence of more complete and articulated specimens. Indeed, the *Auroraphoca atlantica* humerus USNM 250290 is articulated with a partial scapula and an ulna. However, the specimen is clearly juvenile, which we consider inappropriate for holotype designation.

**Description and comparison**

**Figure 8.**
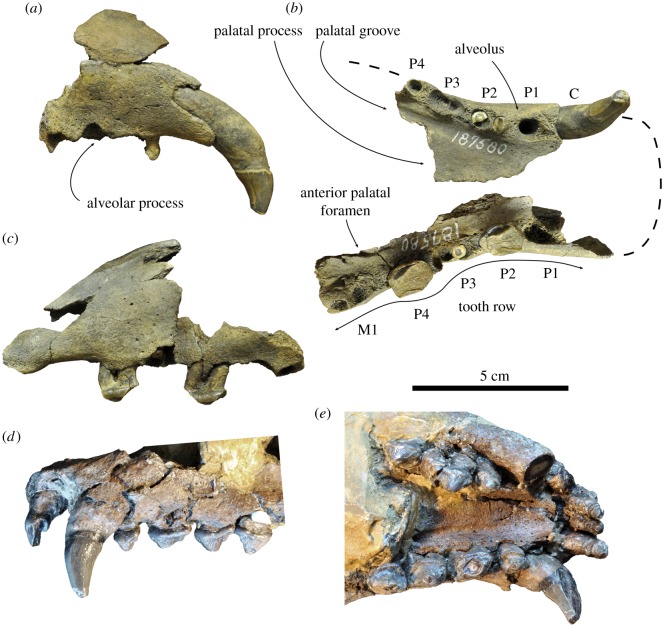
Partial snout USNM 205397 of an indeterminate monachine seal in (*a*) right, (*b*) ventral, and (*c*) left view, formerly considered to represent *Pliophoca etrusca* from the Lee Creek Mine, Aurora, Beaufort County, North Carolina, USA [4]. The fractured holotype snout MSNUP I-13993 from *Pl. etrusca* of Italy, in (*d*) left and (*e*) ventral view. Scale bar equals 5 cm. Image courtesy for *Pl. etrusca*: G Bianucci.

***Maxilla and maxillary postcanines*** (figure 8*a–c*)—Koretsky & Ray [4] assigned 16 isolated maxillae, of which some include maxillary teeth, and eight isolated maxillary teeth from different localities in North Carolina and Florida to *Pliophoca etrusca*. For the original material of *Pl. etrusca* from Italy, i.e. the holotype, no mandibles or mandibular teeth are known [12]. In the absence of articulated specimens having both mandibles and mandibular teeth as well as bones that are comparable to the holotype specimen, it remains impossible to assign isolated mandibles and mandibular teeth to the taxon. The maxilla, on the other hand, is known from the holotype of *Pl. etrusca* and can be compared with specimens from North America; although they are very incomplete.

The state of preservation of the referred and illustrated specimens from North America inhibits a detailed description. The postcanine tooth row is positioned on a lowly raised alveolar process. The postcanine tooth row is slightly diverging posteriorly, as in *Homiphoca*. However, the incomplete state of preservation of the holotype cranium of *Pl. etrusca* from Italy inhibits comparison between the referred specimens and the *Pl. etrusca* holotype. The alveoli for the maxillary teeth in specimen USNM 205397 show that P1 is single-rooted, while the other postcanine teeth are double-rooted. Spacing between the alveolus of P1 and C and P2 is large and shows the presence of a diastema.

On the ventral surface of maxilla USNM 205397, on the palatal process of the maxilla, there is a broad and shallow groove parallel and close to the tooth row. Posteriorly, this groove terminates into the anterior palatal foramen, located at the level between P4 and M1. This condition strongly differs from *Homiphoca* (narrow, deep groove and termination posterior to the anterior alveolus of M1) and from the holotype of *Pl. etrusca* (shallow groove terminating anterior at the level of the posterior alveolus of P3)

Consequently, although the maxillae are very incomplete, both for the referred specimens from North America and the holotype of *Pl. etrusca*, there are a few differences that set both operational taxonomic units (OTUs) apart from each other. We consider it more conservative to consider the referred specimens from North America as Monachinae indet.

**Figure 9.**
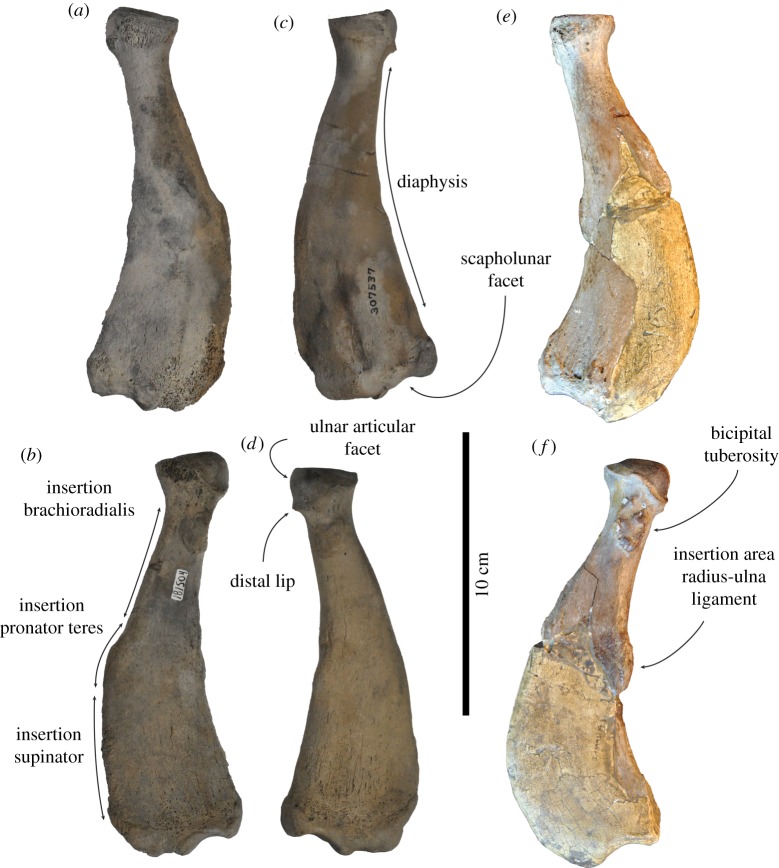
Right radius USNM 181504 in (*a*) lateral, and (*b*) medial view, left radius USNM 307537 in (*c*) lateral, and (*d*) medial view, and formerly considered to represent *Pliophoca etrusca* from the Lee Creek Mine, Aurora, Beaufort County, North Carolina, USA [4]. These specimens differ from the holotype left ulna MSNUP I-13993 from *Pl. etrusca* of Italy, in (*e*) lateral, and (*f*) medial view. Note the incompleteness of the radius of MSNUP I-13993 (*e,f*). Scale bar equals 10 cm. Image courtesy for *Pl. etrusca*: G Bianucci.

***Radius***—Koretsky & Ray [4] assigned 24 radii from the east coast of North America to the species *Pliophoca etrusca* (figure 9*a–d*). One subadult specimen, USNM 250290 has been found associated with a scapula, a humerus and an ulna [4] (see above). However, the radii Koretsky & Ray [4] assigned to *Pl. etrusca* show marked differences with the *Pl. etrusca* holotype radius MSNUP I-13993.

Overall, the radii from North America Koretsky & Ray [4] assigned to *Pl. etrusca* are slender and elongate, with smooth distal expansion of the diaphysis. This condition has also been observed in the holotype radius of *Pl. etrusca*, as well as in the morphotype II radius of *Homiphoca* (see Govender *et al*. [34]). For morphotype I, Govender *et al*. [34, p. 142] argue that ‘the shaft of morphotype I is fairly short and wide with the distal expansion beginning higher on the shaft’; i.e., the distal expansion of the diaphysis of morphotype I is more pronounced in morphotype I of *Homiphoca*. Also, the holotype radius of *Messiphoca mauretanica*, MNHN.F.ORN1, has a short diaphysis that widens very strongly, distally. In lateral view, the curvature of the radii is moderate, intermediate to weakly curved radius of *Homiphoca* and the more strongly curved radius of *Pl. etrusca*. On the proximal epiphysis, the radii which Koretsky & Ray [4] assigned to *Pl. etrusca* bear a well-marked articular facet for the ulna, with a strongly developed distal lip on the posterior margin. This condition has also been observed in *Homiphoca* and *Pl. etrusca*, and only to a lesser extent in *M. mauretanica*. The bicipital tuberosity is subcircular and lowly raised, contrasting to *Homiphoca* and *Pl. etrusca* in which it is rather ovoid and well raised (see Berta *et al*. [12]). The bicipital tuberosity is separated from the proximal epiphysis and the position on the diaphysis varies, being located either medially (USNM 307537) or posteriomedially (USNM 181504) on the diaphysis. Among other Phocidae, all extinct Monachinae, including *Homiphoca*, and *Pl. etrusca* have a medially located bicipital tuberosity, and extant Monachinae have a posteromedially located bicipital tuberosity [12,22]. On the diaphysis, the considered radii bear a weak rugosity approximately halfway along the posterior margin. This is the insertion area for the radius-ulna ligament and is similarly developed in *Homiphoca*, but very prominent in *Pl. etrusca*. On the anterior margin of the diaphysis, the insertion area for the pronator teres muscle is weakly developed, while the insertion areas for the supinator and brachioradialis muscles are smooth, i.e. well developed. This corresponds with observations in extant Phocinae, but contrasts with both extant and extinct Monachinae, except *Monachus monachus* and *Piscophoca pacifica*. In lateral view, the distal portion of the diaphysis is relatively smooth, as in extant Monachinae, while it possesses relatively well-developed grooves for manual tendons in other extinct Monachinae, including *Homiphoca* and *Pl. etrusca*. The scapholunar facet on the distal extremity of the radius is concavoconvex, as in other Phocidae.

The referred radii of Koretsky & Ray [4] share a number of characteristics with *Pl. etrusca*, and Monachinae in general. However, it should be clear that there are numerous differences between the referred humeri and the holotype radius MSNUP I-13993 of *Pl. etrusca*. Consequently, it is highly doubtful that the referred radii from North America can be assigned to *Pl. etrusca*, but that they should rather be considered as Monachinae indet.

**Figure 10.**
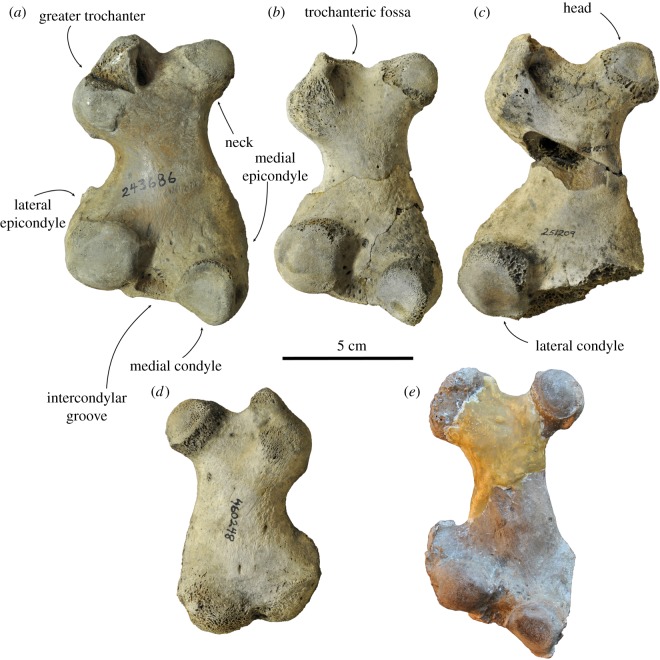
Adult left femora USNM 243686 (*a*), USNM 250293 (*b*), USNM 251209 (*c*), and juvenile right femur USNM 460248 (*d*), in posterior view, formerly considered to represent *Pliophoca etrusca* from the Lee Creek Mine, Aurora, Beaufort County, North Carolina, USA [4]. These specimens are considerably different from the holotype left femur MSNUP I-13993 from *Pl. etrusca* of Italy in posterior view (*e*). Scale bar equals 5 cm. Image courtesy for *Pl. etrusca*: G Bianucci.

***Femur*—**Koretsky & Ray [4] assigned 17 femora from the east coast of North America to *Pliophoca etrusca* (figure 10*a–c*). One specimen, USNM 374222, includes an apparently associated humerus and femur. All other femora from North America that have formerly been assigned to *Pl. etrusca* constitute isolated specimens. Although the holotype femur of *Pl. etrusca*, MSNUP I-13993, is only partially preserved, it is clearly distinct from the ‘*Pl. etrusca*’ femora described by Koretsky & Ray [4]. Both adult (figure 10*a–c*) and juvenile (figure 10*d*) specimens from North America have been considered, judging by the degree of epiphyseal fusion [53]. It can also be noted that there are noticeable differences among the different specimens that Koretsky & Ray [4] consider for *Pl. etrusca*: e.g. the greater trochanter is much broader but lower in USNM 243686 (figure 10*a*) than in the other illustrated adult specimens (figure 10*b,c*). Considering the absence of a detailed quantitative study on the femora of Phocidae, it is yet unknown if the latter variation can be considered intra- or interspecific.

All femora are typically pinniped in that they are longitudinally short and transversely broader than they are anteroposteriorly wide, resulting in a strongly elliptical transverse cross section. In the considered specimens, the minimum diaphyseal width of the shaft is in the proximal portion. Berta *et al*. [12] presented the same condition for the holotype femur of *Pl. etrusca*. However, we disagree and consider the minimum diaphyseal width of the shaft in both *Homiphoca* and *Pl. etrusca* rather in the middle portion of the bone. In the specimens described by Koretsky & Ray [4], the distal epiphysis is wider than the proximal epiphysis in anterior and posterior view; similar to the condition in *Homiphoca* and *Pl. etrusca*. Although the femoral head is not completely preserved, it is hemispherical, as in *Homiphoca* and *Pl. etrusca*. On the contrasts, the neck does not appear as strongly marked as in *Homiphoca* and the holotype of *Pl. etrusca*. The greater trochanter of the specimen USNM 243686 is only lowly raised and transversely broad, contrasting to the other illustrated specimens from North America, as well as the holotype of *Pl. etrusca*, in which the greater trochanter is transversely less broad, but more highly raised. In all specimens from North America, the greater trochanter bears a distinctive trochanteric fossa. While this trait is shared among all Phocinae, such a trochanteric fossa is rarely present among Monachinae (including *Pl. etrusca*), only the extant *Lobodon carcinophaga*, and the extinct *Homiphoca* and *Piscophoca pacifica*. The femora that have formerly been assigned to *Callophoca obscura* also have a well-developed trochanteric fossa.

In anterior view, the diaphysis of the femur gradually widens distally into the distal epiphysis. As a result, the distal portion of the femur attains a strongly triangular appearance. This corresponds with the general condition among Monachinae (L. Dewaele 2017, personal observation). Dewaele *et al*. [11] also noted a strongly triangular distal part in the femur of the phocine *Prophoca rousseaui*. Yet, generally, the distal portion of the diaphysis widens abruptly in Phocinae. The distal portion of the holotype femur of *Pl. etrusca* attains a rather phocine appearance. The epicondylar crest is lowly raised and robust, gradually merging into the diaphysis, proximally (distinct thin ridge in *Pl. etrusca*). The lateral epicondyle is weakly developed and smoothly tapers into the diaphysis, proximally; while the lateral epicondyle is much more prominent in *Pl. etrusca*, recurving proximally. The lateral condyle is large and rounded and the medial condyle is correspondingly large. Both condyles are widely spaced and separated by a wide intercondylar groove. The patellar facet is wider than it is high, as is widely observed among Monachinae [22].

Overall, the femora which Koretsky & Ray [4] identified as *Pl. etrusca* from North America show little similarity with the holotype femur of *Pl. etrusca*. Consequently, their designation as *Pl. etrusca* is contested. Nevertheless, because we advocate for a more conservative approach to specimen designation in this study, we reassign the femora from North America that have formerly been identified as *Pl. etrusca*, to Monachinae indet. It should be noted that these femora are strongly similar to each other and may belong to the same taxon, and that they superficially resemble the femora that have formerly been assigned to *C. obscura*. However, as stated above, we limit the current fossil record of *C. obscura* to the lectotype humerus and other humeri.

## 4. Discussion

The fossil record of all extinct Monachinae taxa that have been described from the Neogene of the North Atlantic Ocean (including the Mediterranean Sea) has been reassessed (table 2). As shown from the descriptions of the different taxa and specimens, monachine humeri are generally easily distinguishable and may be considered as an acceptable basis for identifying different Monachinae. However, it should be clear that this requires (nearly) complete specimens. Hence, both species of *Terranectes* are considered nomina dubia: the holotype of *Terranectes magnus* is a very incomplete humerus, and the holotype of *Terranectes parvus* is a femur and cannot be compared with most other Monachinae from the North Atlantic realm. In the absence of a more complete fossil record, i.e. with more complete and articulated specimens, we strongly advocate the consistent use of at least nearly complete humeri (and associated or articulated bones) as type specimens of fossil phocid taxa. The noticeable intraspecific variation observed, as well as the interspecific similarities on some parts of the humerus support our assertion that incomplete specimens are insufficiently diagnostic for species identification.

**Table 2.** Late Miocene and Pliocene Monachinae from the (North) Atlantic realm, including the Mediterranean Sea. With indication of the respective region where specimens of each taxon are known from.

taxon	synonymy	revised validity	region
*Auroraphoca atlantica*			northwest Atlantic
*Callophoca obscura*	*Mesotaria ambigua*		northeast and northwest Atlantic
*Homiphoca capensis*	*Prionodelphis capensis*		southeast Atlantic
*Homiphoca* sp.			southeast and northwest Atlantic
*Messiphoca mauretanica*		species inquirenda	Mediterranean Sea
*Pliophoca etrusca*			Mediterranean Sea
*Terranectes* spp.		nomen dubium	northwest Atlantic
*Virginiaphoca magurai*			northwest Atlantic

The question about the validity of *Messiphoca mauretanica* is more complex: the holotype humerus MNHN.F.ORN1 is only partially preserved; however, it is associated with an ulna, a radius and vertebrae. This entails a more complete type specimen than for many other phocids, but ulnae, radii and vertebrae are rarely articulated or associated with type specimens in other Neogene Monachinae from the North Atlantic, precluding comparison (see above). Consequently, we consider it appropriate to leave *M. mauretanica in limbo*, pending the discovery of new specimens prior to further investigation regarding the validity of this species.

Comparison of the fossil monachine fauna from the late Miocene and Pliocene of Europe (east Atlantic) with that from the late Miocene and Pliocene of the east coast of North America (west Atlantic) reveal strongly different biogeographic patterns than have previously been proposed. Koretsky & Ray [4] suggested a strong connection between both sides of the North Atlantic, considering the co-occurrence of *Callophoca obscura* and *Pliophoca etrusca* on both sides. However, in light of our taxonomic revision, this is called into question. The absence of *Pliophoca* in North America and the presence of two newly described species that are currently only known from the east coast of North America suggest that there was little ‘interchange’ between monachine faunas on the east coast of North America and the North Sea Basin (and South Atlantic, when including *Homiphoca*). However, the temporal differences between the late Miocene and Pliocene fossil phocid-bearing strata from the North Sea Basin, the east coast of North America, and the Mediterranean, joined by the incomplete fossil record of both sides of the North Atlantic, with many taxa known from exceedingly fewer specimens, precludes being conclusive on the question of the palaeodiversity of phocids in the North Atlantic during the late Neogene.

The current fossil record indicates that there was little faunal interchange between the seal faunas from the east coast of North America and the North Sea Basin, with *C. obscura* being the only monachine seal recorded at both sites. The fossil record of Monachinae from the Neogene of the Mediterranean is small [6,7,12,15,35,54–56] and primarily consists of very fragmentary material [6,35,54–56]: the skull of *Monotherium gaudini* from the late Oligocene–early Miocene of Italy [7,50], *Afrophoca libyca* from the Burdigalian of Libya [6], indeterminate Monachinae from the middle Miocene of Italy and Malta [54–56], and *Pliophoca etrusca* and *Pliophoca* cf. *Pl. etrusca* from the late Pliocene of Italy. This precludes drawing detailed conclusions on palaeobiogeographic changes. However, the fossil record suggests the apparent absence of any late Miocene–early Pliocene Monachinae in the Mediterranean realm, while Monachinae were diverse in the North Atlantic during that time interval. On the contrary, *Pl. etrusca* was present in the Mediterranean realm during the late Pliocene, while Monachinae seem to have disappeared from the North Atlantic at that time.

*Pliophoca etrusca* is widely considered to be a sister-taxon to the modern monk seals of the genus *Monachus* [12], phylogenetically supporting a Mediterranean origin of the monk seals. The research presented in the current study shows a higher diversity of Monachinae from the east coast of North America than previously expected, corroborating Berta *et al*.'s [12] alternative hypothesis of a western North Atlantic origin for the genus *Monachus*. This hypothesis assumes that the genus *Monachus* originated on the east coast of North America and ‘monachinine ancestral stock then presumably dispersed in two directions: (1) to the Mediterranean evolving into *Pliophoca etrusca*; and (2) to the Pacific, which led to the evolution of *Neomonachus schauinslandi* in Hawaii before close of the Panama Seaway (about 3 Ma)’ [12]. Both hypotheses depend on the discovery of more complete specimens of late Neogene Monachinae from the North Atlantic in order to perform phylogenetic analyses and to create a solid basis for morphological arguments. However, recent molecular studies by Scheel *et al*. [57] led to the separation of the three monk seals into two distinct genera, with the Mediterranean monk seal remaining *Monachus monachus*, and the Hawaiian and Caribbean monk seals renamed *N. schauinslandi* and *Neomonachus tropicalis*, respectively. Both genera appear to have been split 6.3 Ma, while *N. schauinsland*i and *N. tropicalis* diverged approximately 3.67 Ma, around the time of closure of the Panama Isthmus [57,58].

## 5. Conclusion

After a careful revision of the fossil record of the Monachinae from the North Atlantic, we contradict Koretsky and Ray's [4] observation of *Pliophoca etrusca* on the east coast of North America during the late Neogene, and we describe two new taxa, from North America. This leads us to conclude that similarities between the Monachinae faunas on both sides of the Atlantic Ocean were actually less than previously expected, with *Callophoca obscura* being found on the east coast of North America and in the North Sea Basin, and with *Homiphoca* sp. from the east coast of North America and Cape Province in South Africa (table 2) and the monachine fauna from North America suggests less faunal interchange than previously assumed.

Formerly, the vast majority of the fossil Monachinae (and by extent Phocidae in general) from the Neogene of the North Atlantic was composed of isolated bones. Most notably, the species *C. obscura* included thousands of isolated bones in private and public collections that are not at all comparable with the isolated lectotype humerus. Our study strongly advocates ending the formerly common practice to use such a syntypic approach to group isolated and incomparable specimens as it is based on weak and untestable arguments. We strongly encourage researchers only to consider complete or nearly complete humeri as potential type specimens for fossil Monachinae, in the absence of cranial specimens or more complete material.

## Supplementary Material

Dinoflagellate cyst biostratigraphy

## Data accessibility

All data for this paper is available within this paper or as electronic supplementary material of this paper. Dinoflagellate cyst biostratigraphy results are provided as electronic supplementary material. This published work and the nomenclatural acts it contains have been registered in ZooBank. The LSID for this publication is urn:lsid:zoobank.org:pub:D9F119E9-64FB-48E8-8509-4D1EFCE0B6C9.
