# Supplementary material for: Diversity of late Neogene Monachinae (Carnivora, Phocidae) from the North Atlantic, with the description of two new species
Source: R Soc Open Sci. 2018 Mar 7;5(3):172437. doi: 10.1098/rsos.172437 (PMC5882749; doi:10.1098/rsos.172437)
Supplement: Dinoflagellate cyst biostratigraphy [file rsos172437supp1.docx]

**Supplementary Information: Dinoflagelate cyst biostratigraphy**

Two sediment samples (sample L15-1105 from the large (potential male) humerus of *Callophoca obscura*, IRSNB M1156-M177, originally described as *Mesotaria ambigua* and sample L15-1108 from a humerus that has formerly been assigned to *C. obscura*, but is currently considered Monachinae indet. (IRSNB 1214) recovered from bone cavities were palynologically analysed for organic-walled dinoflagellate cysts (dinocysts) and acritarchs. The palynological preparation of the sediments followed standard techniques described by Louwye et al. [1]. Acid treatments with HCl and HF were applied for the removal of carbonates and silicates, respectively. Sieving of the organic residue was carried out on a nylon screen with a 10 μm mesh size. The residue was placed on glass slides with glycerol gelatine jelly. The microscopic analysis was carried out with a transmitted light microscope Zeiss AxioImager A1 under a 400x magnification. The entire slide was scanned in non-overlapping traverses. The taxonomy of the dinocysts and acritarchs follows Fensome et al. [2].

For sample L15-1105, the presence of *Barssidinium pliocenicum* indicates an age no older than late Miocene [3]. *Habibacysta tectata* and *Achomosphaera andalousiensis* are recorded and both species have a lowest occurrence in the middle Miocene, respectively at 14.2 Ma and 13.2 Ma [4–6] and occur throughout the upper Miocene. The sample holds furthermore the two biostratigraphical key species *Labyrinthodinium truncatum* and *Selenopemphix armageddonensis*, albeit in low numbers and recorded outside the systematic counting. *L*. *truncatum* has a highest occurrence near the upper boundary of the *Hystrichosphaeropsis obscura* zone. The age of the *H*. *obscura* zone is late Tortonian (8.8 Ma - 7.6 Ma). *S*. *armageddonensis* has a lowest occurrence at the lower boundary of the superjacent eponymous zone and has a latest Tortonian - earliest Zanclean age (7.6 Ma - 5 Ma). Both latter species normally do not occur together, and therefore reworking of sediments of the *H*. *obscura* zone is suspected. A Messinian age can be proposed for the sediment sample.

Given the absence of *Palaeocystodinium* species and *Cleistosphaeridium placacanthum* - both with a hightest occurrence at the upper boundary of the *Amiculosphaera umbracula* zone at 8.8 Ma - reworking of older Tortonian elements can probably be excluded.

For sample L15-1108, the presence of *Barssidinium pliocenicum* indicates an age of the sample not older than late Miocene (Louwye 2002; De Schepper et al. 2004). *Habibacysta tectata* and *Achomosphaera andalousiensis* are recorded and both species have a lowest occurrence in the middle Miocene, respectively at 14.2 Ma and 13.2 Ma [4–6] and occur throughout the upper Miocene. The sample holds furthermore the two biostratigraphical key species *Labyrinthodinium truncatum* and *Selenopemphix armageddonensis*. *L*. *truncatum* has a highest occurrence near the upper boundary of the *Hystrichosphaeropsis obscura* zone of Dybkjaer and Piasecki [4]. The age of the *H*. *obscura* zone is late Tortonian (8.8 - 7.6 Ma). *S*. *armageddonensis* has a lowest occurrence at the lower boundary of the superjacent eponymous zone, and has a latest Tortonian - earliest Zanclean age (7.6 - 5 Ma). Both latter species normally do not occur together, and therefore reworking of sediments of the *H*. *obscura* zone is suspected. The presence of *Palaeocystodinium* species obscures furthermore the biostratigraphic interpretation, since this species has a highest occurrence at the upper boundary of the older *Amiculosphaera* *umbracula* zone at 8.8 Ma. This implicates that sediments of both the *A*. *umbracula* and the *H*. *obscura* zones are reworked into the *S*. *armageddonensis* zone.

**References**

1. Louwye S, Head MJ, De Schepper S. 2004 Dinoflagellate cyst stratigraphy and palaeoecology of the Pliocene in northern Belgium, southern North Sea Basin, *Geological Magazine*, **141**, 353-378. (doi:[10.1017/S0016756804009136](https://doi.org/10.1017/S0016756804009136))
2. Fensome RA, MacRae RA, Williams GL. 2008 DINOFLAG2, Version 1. *American Association of Stratigraphic Palynologists*, Data Series No. 1.
3. Louwye S. 2002 Dinoflagellate cyst biostratigraphy of the Upper Miocene Deurne Sands (Diest Formation) of northern Belgium, southern North Sea Basin. *Geological Journal* **37**, 55-67. (doi:10.1002/gj.900)
4. Dybkjaer K, Piasecki S. 2010 Neogene dinocyst zonation of the eastern North Sea Basin, Denmark. *Review of Palaeobotany and Palynology* **161**, 1-29. (doi:10.1016/j.revpalbo.2010.02.005)
5. Quaijtaal W, Donders T, Persico D, Louwye S. 2014 Characterizing the middle Miocene Mi-events in the Eastern North Atlantic realm: a first high-resolution marine malynological record from the Porcupine Basin. *Palaeogeography, Palaeoclimatology, Palaeoecology* **399**, 140-159. [(doi:10.1016/j.palaeo.2014.02.017](https://doi.org/10.1016/j.palaeo.2014.02.017))
6. Schreck M, Matthiesen J, Head MJ. 2012 A magnetostratigraphic calibration of Middle Miocene through Pliocene dinoflagellate cyst and acritarch events in the Iceland Sea (Ocean Drilling Program Hole 907A). *Review of Palaeobotany and Palynology* **187**, 66–94. [(doi:10.1016/revpalbo.2012.08.006](https://doi.org.10.1016/revpalbo.2012.08.006))
